# A study of the limitations of musical experience in Ancient Chinese Poetry – The case of the creation concert of Wei’s Music Score

**DOI:** 10.1371/journal.pone.0346421

**Published:** 2026-06-11

**Authors:** Zushun Zhang, Panjie Xie, Jinsong Wang

**Affiliations:** 1 School of Music, Anhui University of Arts, Hefei, Anhui, China; 2 School of Design, Jiangnan University, Wuxi, Jiangsu, China; Chengdu Normal University, CHINA

## Abstract

Ancient poetry, with profound artistic and literary value, holds a prominent position among China’s vocal works. Against the backdrop of vigorously promoting China’s outstanding traditional culture in the new era, it has witnessed a revival in interpretations both domestically and internationally. At present, ancient poetry faces dissemination challenges including constraints in performance environments and limitations in historical comprehension. These experiential restrictions impede recipients’ cultural identity, cultural confidence and cultural inheritance. This study adopts a literature review methodology, integrating the conceptual framework of leisure constraints and tourism research. It conducts systematic research on ancient poetry concerts to develop a scale measuring the limiting factors of ancient poetry experience. Using this scale, the study investigates and analyzes statistical results from three Weishi Yuepu (Wei Dynasty Music Score) creative concerts through the PLS-SEM method. Results indicate that constraints on ancient poetry experience exhibit a three-factor structure comprising personal constraints, interpersonal constraints, and structural constraints. This confirms the sequential pathway of ancient poetry experience: Preference→Participation; Personal Constraint→Preference; Structural Constraints→Participation. The study extends the application of the leisure restrictions model to traditional music, benefiting the domestic and international dissemination of China’s outstanding traditional culture while enhancing recipients’ understanding and interest in ancient poetry.

## Introduction

Ancient poetry is a brilliant chapter in traditional culture, fusing literature and music and encapsulating ancestors’ noble sentiments. These works are preserved via ancient Chinese and guqin notation (such as shengquzhe, gongdiao pu, suzi pu, and gongche pu),including classical poems, ci lyrics, and qu songs [1]. At UNESCO headquarters, President Xi Jinping emphasized, “Let museum artifacts, heritage, and ancient texts come alive. Let Chinese and global civilizations offer spiritual guidance and motivation to humanity” [2]. Ancient poetry performances bring new life to texts through verse – history dialogue. The details of a successful ancient poetry concert are not randomly designed or isolated; instead, they form poetic presentations based on musical purpose and aesthetic principles.

Ancient poetry faces challenges in contemporary creative performance, including difficulties in comprehension, appreciation, transmission, and dissemination. Current academic research primarily focuses on collecting musical scores and cultivating performers, emphasizing “primary creation” and “secondary creation” while neglecting “tertiary creation”, hindering its widespread dissemination [3,4]. By exploring the experiential constraints of “tertiary creation” and integrating Leisure Constraints theory, this study proposes the following hypotheses: Personal Constraints→ Preference→Negative; Interpersonal Constraints→Participation→Negative; Structural Constraints→Participation→Negative; Interpersonal Constraints→Satisfaction→Negative; Structural Constraints→Satisfaction→Negative. Meanwhile, it puts forward supplementary hypotheses: Preference→Participation→Positive; Participation→Satisfaction→Positive. Using three original concert cases of ancient poetry set to music from the Wei Dynasty Score, combined with Partial Least Squares Structural Equation Modeling (PLS-SEM) analysis, this study aims to offer new perspectives for researchers on the perception, participation and satisfaction associated with “triple creation”. It seeks to explore innovative approaches to inheriting and promoting traditional poetry culture, arouse public interest in traditional culture, and enhance the prominence of traditional Chinese culture both at home and abroad.

## Literature review

Current research on ‘Leisure restrictions’ both domestically and internationally has expanded beyond superficial observations to explore underlying mechanisms and diverse datasets. The scope of research subjects and content is broad, emphasizing the integration of quantitative and qualitative approaches, as well as theoretical and empirical methodologies. Early studies predominantly comprised conceptual review articles [5,6]. Crawford D W [7], Jackson E L, and others examined constraints within hierarchical relationships (intrapersonal constraints, interpersonal constraints, structural constraints).

Although constraint theory was proposed as early as the 1960s and 1970s, it was only recently that leisure restrictions theory was introduced into tourism research. Researchers have examined tourism constraints across diverse sectors, including nature tourism [8,9], museum tourism [10], event tourism [11,12], sports tourism [13–15]. In the arts sector, Zhang Honglei and Zhang Jie examined constraints on experiencing traditional Chinese cultural landscapes (calligraphy landscapes) [16]; Guo Yunjiao et al. studied leisure restrictions and activity benefits among low-income workers participating in artistic activities [17]. In the sports sector, Zhou Liangjun et al. applied the leisure constraints model in marathon participation behavior research, identifying structural constraints as the primary barrier to marathon participation [18]; Jin Qingyun focused on exploring the relationship between leisure restrictions and participation in recreational sports among urban residents in ethnic minority areas of Yanbian [19]. During extensive literature review, it was found that the majority of research subjects were female [20,21]. Research on adolescents [22–24] and older adults [25,26] is also prevalent. Additionally, studies on leisure restrictions encompassed case studies involving individuals with disabilities [27] and children [28]. Regarding leisure categories, most journals focused on questionnaire data analysis for sports, painting arts, and environmental arts. Research on music performance categories lacked systematic literature support, particularly concerning ancient poetry—a category of historical significance—which had insufficient case illustrations within the leisure restrictions field. Therefore, this study on the creation and performance of ancient poetry in Mouding employs leisure restrictions theory for in-depth analysis. It aims to propose innovative insights, explore participants’ cognitive attitudes toward ancient poetry, and thereby effectively enhance participant satisfaction and engagement. Based on the definition of leisure constraints, constraints on ancient poetry experiences can be defined as factors that inhibit continued engagement with ancient poetry. These factors prevent participation in ancient poetry activities, hinder the maintenance or increase of participation frequency, and may negatively impact the quality of ancient poetry experiences.

The scale development process rigorously followed Churchill’s (1979) paradigm, focusing on an initial assessment of content and face validity prior to formal data collection [29]. The initial item pool consisted of 22 items. Most of the items were adapted from established leisure constraints literature [15,30], and a small subset was newly developed specifically to capture the context of ancient Chinese poetry experiences [31,32]. To ensure robust content validity, an expert panel comprising three scholars specializing in traditional Chinese musicology and tourism management reviewed the initial pool.

Furthermore, to ensure the phrasing was clear and unambiguous, a preliminary small-scale pre-test was conducted with a convenience sample of 15 participants. This step focused purely on qualitative face validity and readability, with participants asked to provide verbal or written feedback on whether any items were confusing or difficult to understand. The explicit decision rules for item deletion at this stage were qualitative rather than statistical: items were flagged for removal if the expert panel deemed them semantically redundant or if pre-test respondents reported cognitive ambiguity. Based on this expert consensus and qualitative pre-test feedback, two items exhibiting weak contextual relevance were removed. No quantitative dimension reduction or statistical hypothesis testing was performed during this preliminary stage. The final 20-item scale was then deployed in the formal survey, where its internal consistency and convergent validity were subsequently confirmed via PLS-SEM analysis.

H1.1: Personal internal constraints negatively impact participants’ preferences.
H1.2: Interpersonal relationship constraints negatively affect participants’ engagement.
H1.3: Structural constraints negatively affect participants’ engagement.
H1.4: Interpersonal relationship constraints negatively impact participants’ satisfaction.
H1.5: Structural constraints negatively impact participants’ satisfaction.

**Fig 1:**
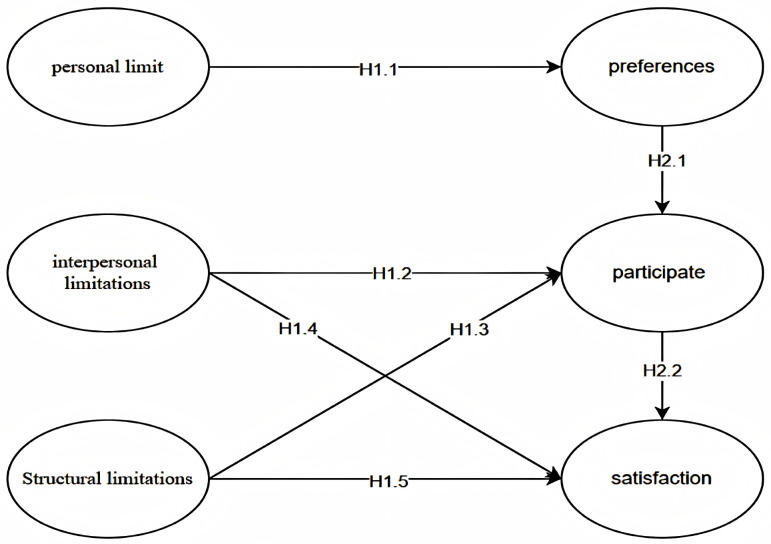
Restriction model of ancient poetry.

Based on leisure restrictions theory and Walker, Halpenny, Spiers, and Deng’s (2011) [33] research, preferences may predict participation, and participants may experience greater satisfaction when actively engaged in an activity. Therefore, we can propose two additional hypotheses (Fig 1).

H2.1: Preferences are positively correlated with participation.
H2.2: Engagement is positively correlated with participant satisfaction.

## Materials and methods

### Research locations and methods

The experiment was conducted through three ancient poetry concerts hosted by the Center for the Inheritance and Innovation of Ancient Chinese Music and Poetry at Anhui University of Arts. One event, the closing ceremony of the Ancient Poetry Inheritance Workshop on the Wei Family Music Score, was organized by relevant departments and the university. It attracted over 100 participants from multiple provinces and 200 members of the public. Participants registered via email, and no personal – info screening was done until the 100 – person capacity was reached. Attendees were from diverse fields. The 200 concert – goers came after seeing announcements on the official account, and admission was free. The concert had guided listening to introduce classical poetry, and with tech elements, workshop participants recited songs corresponding to AI – processed poetry audio for a light particle display, creating an immersive multimedia experience. 277 paper questionnaires were distributed, 223 were returned and compiled for accuracy. All procedures in this study complied with institutional research guidelines and the 1964 Declaration of Helsinki and its subsequent amendments or equivalent ethical standards. Survey responses were collected anonymously via Qwenzhixing and paper questionnaires. Informed consent was obtained from all respondents and their guardians upon questionnaire distribution.

The center collaborated with the Anhui Museum and the Hefei Musicians Association to host two “Ancient Melodies, Modern Voices” ancient poetry concerts. Three hundred audience members were randomly selected via public account reservations to attend each live performance, and the process was open to the public without system – based selection. Through innovative arrangements, ancient poetry was integrated with historical and cultural elements. Each concert combined immersive experiences with the Anhui Provincial Museum’s “Capital of the World” exhibition and the 2024 Cultural Heritage Interpretation Showcase Final Selection. This explored the contemporary value of ancient poetry and showcased the charm of Ming Dynasty musical relics, including the “Wei’s Musical Score”.

Before the concert, audiences can join a guided tour of the museum’s “Capital of the World” exhibition with artifacts from the Ming Dynasty’s Hongwu period to explore Ming culture. Through this experience, attendees will appreciate the musical beauty of ancient poetry and understand its historical and cultural context, strengthening the connection to traditional heritage. The concert features six sets of ancient poetry songs selected and arranged by the Center for Inheritance and Innovation of Ancient Chinese Music and Poetry at Anhui Arts University, which are related to Anhui’s heritage. Each set’s cultural essence is presented through commentary, piano, and ethnic chamber music. After the concert, paper and electronic questionnaires were distributed to collect audience feedback for future events. Survey results showed strong audience approval for the history – music fusion, with many looking forward to similar experiences. To ensure comprehensive feedback from students and the elderly who may not be good at mobile scanning, a combined approach of paper and electronic questionnaires was used for future event planning.

### Data acquisition and survey sample analysis

The questionnaire for this study primarily includes the following components: a measurement scale for limiting factors in ancient poetry experiences, a scale for measuring preferences, personal constraints, and satisfaction, as well as demographic characteristics and traits of participants. The scale for measuring constraints on ancient poetry experiences employed a 5-point Likert scale: 1 indicated strongly disagree (dissatisfied), 2 indicated disagree (dissatisfied), 3 indicated neutral, 4 indicated agree (satisfied), and 5 indicated strongly agree (satisfied). Data collection occurred during three ancient poetry concerts held between August and December 2024. To ensure sample openness and diversity, audiences for the latter two concerts were publicly recruited via the official WeChat public account using a first-come-first-served random reservation model. Each concert automatically closed registration upon reaching 300 attendees, eliminating human selection bias. To accommodate audience habits across age groups—particularly avoiding a digital divide for students and seniors—data collection employed a hybrid approach combining paper and electronic questionnaires. Across the three survey phases, 322 paper questionnaires were distributed with 281 returned, while 226 electronic questionnaires were simultaneously collected. After rigorous quality control of all returned data—including deduplication based on demographic information, response time screening, and exclusion of questionnaires with duplicate selections—411 valid questionnaires were obtained. The overall response rate and validity rate were 92.5% and 75%, respectively.

Furthermore, to ensure the adequacy of the sample size for PLS-SEM analysis [34], this study applied the widely recognized ‘10-times rule’ [35]. In the proposed structural model, the maximum number of structural paths pointing to a single endogenous construct is three (e.g., Participation is predicted by Preference, Interpersonal Constraints, and Structural Constraints). Consequently, the minimum required sample size under this guideline is 30. The final valid sample of 411 obtained in this study significantly exceeds this requirement, ensuring sufficient statistical power and robustness for model estimation.

### Descriptive statistics of the sample

This study collected data related to demographic characteristics. Overall, the research obtained high-quality data through offline questionnaire surveys. The sample encompassed individuals of different genders, ages, income levels, occupations, educational backgrounds, and musical foundations, demonstrating strong representativeness and validity. The data is divided into six age groups: 56.91% are aged 18–45, 31.9% are under 18, and 0.48% are over 65. The gender ratio is 33.1% male and 66.9% female. By occupation, students and professionals constitute the largest groups, accounting for 43.31% and 24.09% respectively. Regarding educational attainment, 60.34% held bachelor’s degrees or higher, with 21.17% holding master’s degrees or higher. The rate of meeting basic musical proficiency standards was 71.78%, while science and engineering backgrounds accounted for 28.22%. The above data reveals overall trends and characteristics of the group in terms of age, gender, occupation, education, and musical skills: the occupational distribution exhibits knowledge-intensive features, and musical literacy shows significant correlations with educational background and professional field. This structural characteristic holds significant reference value for analyzing the reasons behind the leisure restrictions of ancient poetry (Table 1).

**Table 1:** Descriptive statistics.

Variable	Category	Frequency	Proportion
Age	Under 18 years of age	131	31.9
	19-24	48	11.67
	25-30	59	14.35
	31-35	47	11.43
	36-45	80	19.46
	46-55	28	6.81
	56-64	16	3.89
	65 and over	2	0.48
Gender	Male	136	33.1
	Women	275	66.9
Occupation	civil servant	66	16.06
	Enterprise and institutional managers	46	11.19
	Professional/Technical and Educational Personnel	99	24.09
	Service Sales and Trade Personnel	5	1.22
	Others	2	0.49
	student	178	43.31
	Retirees	15	3.65
Educational attainment	Primary and below	28	6.81
	junior high school	8	1.95
	High school and secondary school	96	23.36
	three-year college	31	7.54
	undergraduate (adjective)	140	34.06
	bachelor’s degree	87	21.17
	PhD	21	5.11
Music Fundamentals	Yes	295	71.78
	No	116	28.22

### Research methods

This study employs Partial Least Squares Structural Equation Modeling (PLS-SEM) for analysis, which aligns exceptionally well with the research characteristics for three primary reasons: (1) Research Objectives: A core objective of this study is to predict key endogenous variables (e.g., preferences, personal constraints, and satisfaction). PLS-SEM optimizes predictive capability by maximizing variance explained (R² value) for endogenous latent variables, directly serving this objective; (2) Sample Size and Model Complexity: With a sample size of N = 411 and a model incorporating multiple constructs, this study involves moderate complexity. PLS-SEM demonstrates robustness in handling such complex models when sample sizes are not extremely large; (3) Theoretical Development Stage: This research exhibits exploratory characteristics to a certain extent, and PLS-SEM is highly suitable for such theoretical development and analysis.

All analyses were conducted using SmartPLS 4.0 software [36]. Model estimation employed the partial least squares (PLS) algorithm with the path weighting scheme, converging upon reaching the 10^−7^ termination criterion. To rigorously evaluate the significance of the path coefficients and structural model metrics, we performed a Bootstrap resampling procedure. For full transparency and reproducibility, the exact estimation settings utilized were: 5,000 subsamples generated using the ‘without replacement’ method, with 95% bias-corrected and accelerated (BCa) confidence intervals, and the ‘no sign changes’ option selected (Hair et al., 2019). All reported inferential statistics in this study are derived from these consistent settings.

## Analysis of results

### PLS-SEM analysis results

**Fig 2:**
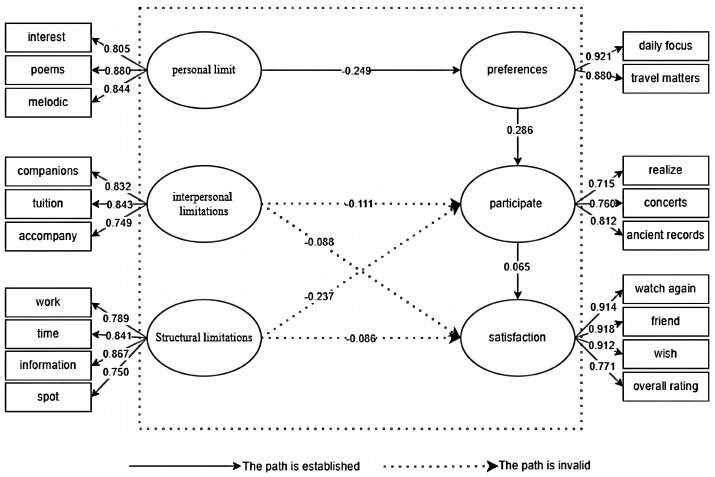
Structural model of ancient poetry.

This study employed Smart-PLS 4 software for data analysis [37,38] and systematically evaluated the reliability and validity of measurement instruments based on the criteria established by Roldan and Cepeda (2017) and Hair et al. (2019) [39]. As shown in Table 2, factor loadings for all measurement items exceeded 0.7. Cronbach’s Alpha coefficients (α > 0.6) and composite reliability (CR > 0.7) jointly confirmed the internal stability of variables. Additionally, the average variance extracted (AVE) for all latent variables surpassed the 0.5 threshold, meeting the fundamental requirement for convergent validity. The VIF values among potential formative variables in each model were all below 5, ruling out multicollinearity issues in model estimation. Overall, the scale demonstrated sound reliability and validity, providing a reliable data foundation for subsequent structural model analysis (Fig 2).

**Table 2:** Convergent validity measures.

Variable	Test items	Factor Load	VIF	Cronbach’s a	CR	AVE
Personal limit				0.798	0.881	0.711
	You don’t like to listen to concerts about old poems, you find them boring.	0.805	1.383			
	Understanding the poetic context of Chinese Ancient Poetry can be difficult for you	0.880	2.370			
	Understanding the musical melody of ancient poems can be difficult for you.	0.844	2.210			
Interpersonal limitations				0.737	0.85	0.655
	No one in your neighborhood is interested in Ancient Score Poetry Music	0.832	1.499			
	There is no one to guide you in understanding and singing the ancient poems.	0.843	1.557			
	Unaccompanied Ancient Poetry Concerts limits the number of times you can watch them.	0.749	1.372			
Structural limitations				0.832	0.888	0.664
	You are less likely to encounter things in your life that are related to the music of ancient sheet music and poetry	0.798	1.952			
	Working and living conditions limit your chances of enjoying a concert of ancient poems and lyrics	0.841	2.180			
	You don’t have a lot of time to enjoy concerts or lectures on ancient music and poetry	0.867	1.941			
	There is not enough information in the society to guide you to enjoy the music of old scores and poems.	0.750	1.551			
	The distance between concerts, salons, and other events limits your participation.			0.771	0.896	0.812
Preferences		0.921	1.647			
	In your daily life, you look out for things with ancient poems (e.g., lectures, concerts, salons, etc.)	0.880	1.647			
	You will be on the lookout for things with ancient poems during your travels (e.g., museum visits, street culture, flash mobs, etc.).			0.640	0.807	0.582
Participate		0.715	1.198			
		0.760	1.276			
	Your level of knowledge of Chinese Ancient Poetry	0.812	1.330			
	Your average number of concerts per year			0.907	0.932	0.776
	The average number of times you listen to classical music concerts per year	0.914	3.395			
Satisfaction		0.918	3.490			
	Would you like to see a concert of ancient scores and poems about Wei’s Music Score again?	0.912	2.746			
	Would you like to recommend “Wei’s Music Score” Ancient Score Poetry Concert to your friends and family?	0.771	2.011			
	Would you like to see a similar concert of ancient music again in the future?			0.798	0.881	0.711
	Your review of the Wei’s Music Score Concert for the Creation of Ancient Score and Poetry	0.805	1.383			

**Note:** CR is the combined reliability; AVE is the variance,VIF is the covariance statistic.

According to the Fornell-Larcker criteria, the discriminant validity of each construct—that is, whether constructs are sufficiently distinct—is determined by comparing the square root of each construct’s average variance extracted (AVE) with its correlations to other constructs. If a construct’s square root of AVE exceeds its correlations with other constructs, it satisfies discriminant validity. In summary, all constructs meet the Fornell-Larcker criteria, indicating they possess good discriminant validity (Table 3).

**Table 3:** Distinctive validity of variables (Fornell-Larcker Standard).

	Personal limit	Interpersonal limitations	Preferences	Participate	Satisfaction	Structural limitations
Personal limit			0.798	0.881	0.711	
Interpersonal limitations	0.805	1.383				0.805
Preferences	0.880	2.370				0.880
Participate	0.844	2.210				0.844
Satisfaction			0.737	0.85	0.655	
Structural limitations	0.832	1.499				0.832

Discriminant validity was assessed using the Heterotrait-Monotrait Ratio (HTMT), a modern and rigorous standard for evaluating construct discrimination. Results are presented in Table 4. All construct pairs yielded HTMT values below the conservative threshold of 0.90 (Henseler et al., 2015), indicating that the measurement model possesses good discriminant validity.

**Table 4:** HTMT evaluation.

Project	Personal constraints	Interpersonal constraints	preference	Participate	Satisfaction	Structural constraints
Personal constraints						
Interpersonal constraints	0.680					
preference	0.313	0.361				
Participate	0.279	0.511	0.544			
Satisfaction	0.298	0.208	0.401	0.180		
Structural constraints	0.531	0.890	0.306	0.514	0.190	

To examine the discriminant validity of the measurement model, this study conducted cross-loadings analysis. As shown in Table 5, all measurement items exhibited higher loadings on their intended target constructs than on any other non-target constructs (i.e., cross-loadings), providing support for discriminant validity.

**Table 5:** Cross-load matrix.

Project	Personal constraints	Interpersonal constraints	Preference	Participate	Satisfaction	Structural constraints	average value	standard deviation
interest	0.805	0.371	−0.231	−0.109	−0.241	0.321	1.976	0.943
poems	0.88	0.453	−0.209	−0.186	−0.187	0.372	2.139	0.963
melodic	0.844	0.488	−0.184	−0.206	−0.212	0.401	2.158	0.968
work	0.372	0.53	−0.213	−0.231	−0.193	0.798	2.764	1.188
time	0.383	0.557	−0.241	−0.299	−0.148	0.841	2.783	1.154
information	0.356	0.679	−0.273	−0.429	−0.111	0.867	3.049	1.17
spot	0.294	0.498	−0.081	−0.273	−0.123	0.75	2.900	1.093
companions	−0.138	−0.247	0.246	0.715	0.124	−0.297	2.384	1.052
tuition	−0.186	−0.217	0.328	0.76	0.144	−0.236	3.068	1.167
accompany	−0.124	−0.348	0.31	0.812	0.043	−0.357	2.596	1.189
daily focus	−0.225	−0.131	0.194	−0.03	0.771	−0.077	3.869	1.378
travel matters	−0.237	−0.265	0.921	0.386	0.301	−0.253	4.212	1.378
realize	−0.211	−0.221	0.88	0.305	0.329	−0.205	2.676	0.891
concerts	0.502	0.832	−0.198	−0.29	−0.193	0.516	2.759	1.041
ancient records	0.322	0.843	−0.229	−0.338	−0.086	0.63	1.903	0.912
overall rating	0.432	0.749	−0.239	−0.233	−0.136	0.57	4.328	0.775
watch again	−0.209	−0.145	0.323	0.141	0.914	−0.134	4.304	0.827
friend	−0.202	−0.157	0.341	0.107	0.918	−0.155	4.294	0.788
wish	−0.266	−0.164	0.322	0.166	0.912	−0.196	4.350	0.754

Path analysis results indicate (see Table 6) that perceived personal constraints significantly negatively predict individuals’ preferences when engaging in ancient Chinese poetry experiences (path coefficient = −0.249, p < 0.001). This finding suggests that when individuals face greater limitations due to time, energy, or other subjective factors, their interest and preference for ancient poetry experiences significantly diminish. Furthermore, preferences positively and highly significantly predict participation behavior(path coefficient = 0.296, p < 0.001). That is, the greater an individual’s interest in ancient poetry experiences, the higher the likelihood of their actual participation. This finding underscores the pivotal role of interest as a driving force in cultural experience behaviors. Simultaneously, structural constraints significantly negatively predict personal constraints.(path coefficient = −0.237, p < 0.001). This indicates that when external conditions—such as accessibility of activity resources, social support, or cultural environment—create high entry barriers, individuals’ willingness to engage in classical poetry experiences is suppressed. Collectively, both personal and structural constraints subtly yet substantially predict individual preferences and participation willingness.Future efforts to promote and disseminate ancient poetry experiences should focus on reducing these subjective and objective barriers to enhance interest levels.

**Table 6:** Analysis of model path effect test.

Effects of endogenous variables	Path coefficient	95% CI	T-value	P values	Support for assumptions
Personal limit - > Preferences	−0.249	[−0.355, −0.156]	4.842	0.000	be tenable
Interpersonal limitations - > Participate	−0.111	[−0.240, 0.016]	1.682	0.093	untenable
Interpersonal limitations - > Satisfaction	−0.111	[−0.240, 0.016]	1.682	0.093	untenable
Preferences - > Participate	0.296	[0.202, 0.389]	6.249	0.000	be tenable
Participate - > Satisfaction	0.296	[0.202, 0.389]	6.249	0.000	untenable
Structural limitations - > Participate	−0.237	[−0.354, −0.121]	4.030	0.000	be tenable
Structural limitations - > Satisfaction	−0.237	[−0.354, −0.121]	4.030	0.000	untenable

In the Partial Least Squares Path Model (SEM-PLS), the R-squared (R²) value serves as a crucial indicator for measuring the extent to which the model explains the variance in the dependent variable. The R² value ranges from 0 to 1, with higher values indicating greater explanatory power of the model. Cohen (1988) [40] defined the boundaries for small effect R² = 0.02, medium effect R² = 0.13, and large effect R² = 0.26. The R² values for personal constraints, satisfaction, and preference all exceed 0.02, indicating that the model possesses partial explanatory power (see Table 7).

**Table 7:** Variance explained.

	R²	Adjusted R2
Participate	0.247	0.242
Satisfaction	0.038	0.031
Preferences	0.062	0.060

Nevertheless, we recognize that the R² values for preference (0.062) and satisfaction (0.038) are relatively low, which suggests that the present model only explains a limited proportion of variance in these two constructs. While our current interpretation is reasonable, such modest explanatory power indicates that some important factors may not have been incorporated into the analytical framework. Accordingly, we acknowledge that potential omitted variables—such as cultural capital and prior poetry exposure—may also exert substantial influences on individuals’ preference and satisfaction, and could account for the remaining unexplained variance. These limitations are duly noted and will be further examined and addressed in future in-depth research.

## Discussion

The above data indicate that personal and structural constraints are relatively significantly correlated with preferences, while interpersonal constraints have a less pronounced effect. Preferences are positively correlated with participation, but the direct impact of participation on satisfaction is relatively weak. This aligns with findings in the calligraphy landscape study regarding how intrapersonal factors correlate with preferences and how preferences affect participation [41]. This suggests that when enhancing participation and satisfaction, we should prioritize individual and structural factors. Meanwhile, don’t overlook the mediating role of preferences, as their facilitative effect on participation is significant. Further analysis shows that personal and structural constraints have significant negative effects, indicating they hinder participation and satisfaction improvement. Moreover, the positive impact of preference on participation emphasizes the importance of intrinsic motivation. Stimulating this motivation can help overcome constraints and increase engagement. At the same time, optimizing personal constraints for ancient poetry performances can improve both participation and satisfaction. As a complex psychological construct shaped by enduring personal factors, satisfaction and preferences inherently differ in ways that cannot be fully captured or predicted by single external situational factors (e.g., the constraints in this model) Nevertheless, the significant path relationships identified in the model reveal stable intrinsic connections among these variables. We acknowledge this as a limitation of the present study and identify it as a direction for future in-depth research.

### Addressing personal constraints to enhance preference values

“Understanding the poetic imagery of ancient poetry is difficult for you” has a factor loading of 0.880 in Personal constraints, indicating that the difficulty of comprehending ancient poetic imagery is a primary factor influencing preference. “Understanding the musical melody of ancient poetry is difficult for you” has a factor loading of 0.844 in Personal constraints, showing that the difficulty of grasping musical melodies also significantly impacts preference. This may stem from ancient poetry often embodying rich historical and cultural connotations, involving essential knowledge of seasonal cycles, geography, and history. It comprises structured language deviating from everyday usage, with poetic genres imposing unique combinatorial constraints on linguistic elements, demanding considerable literary and artistic cultivation from the recipient [42]. To address this, detailed concert optimization strategies can be provided to reduce comprehension difficulty, thereby enhancing user preference for classical poetry.

#### Optimizing concert pathways.

The construction of field spaces is closely related to concert content and form. Bourdieu defines fields as networks of objective relations. Most contemporary poetry performance venues are unidirectional theater – concert halls with physical distance between stages and audience seating, creating hierarchical divisions. Sociologist Christopher Small notes that modern concert halls assume musical performance is a one – way transmission, with the composer’s intent conveyed to the audience via the performer [43]. This unidirectional model limits audience-performer interaction, reducing audience experience. Optimizing field design by shortening distance and increasing interactivity is crucial for better performance. Immersive environments enhance engagement and satisfaction. For example, performers use body language and verbs to add creativity. Costumes symbolize performance, compensating for limited eye contact. Guided listening uses vivid language to help audiences understand poetry. By reimagining landscapes, audiences resonate with ancient rhythms, internalizing culture and appreciating classical verse.

#### Online promotion channels.

Through the official account “Ancient Melodies, Modern Voices,” we provide listeners with a rich and diverse array of online music resources. The account regularly publishes knowledge about ancient poetry, case studies, and event previews to enhance public awareness and interest in classical verse. Simultaneously, collaborations with museums, musicians’ associations, television stations, music radio stations, and other government media outlets further stimulate public enthusiasm for traditional culture. Leveraging interactive features like comments and polls, we gather user feedback and needs to continuously refine promotional content, ensuring it aligns with actual user demands. ntegrating media promotion with offline events creates a synergistic online-offline campaign model that further strengthens the global reach and resonance of ancient poetry. Additionally, utilizing social media platforms such as Weibo and Douyin, we release short videos and livestreams to showcase the charm of ancient poetry and attract younger audiences. Regularly hosting online concerts and poetry recitals featuring renowned artists enhances interactivity and boosts user engagement. Data analysis enables precise targeting of audiences and the development of personalized promotion strategies to maximize dissemination effectiveness. Through multi-channel coordination, ancient poetry gradually integrates into everyday life, revitalizing this artistic tradition with renewed vitality in the modern era.

### Leveraging preference orientation to stimulate engagement

Individuals who report a preference for engaging with ancient poetry–related content (e.g., lectures, concerts, salons) in daily life are significantly more likely to actively participate in poetry-related activities. This stems from the observation that ancient poetry already possesses a dedicated following from universities, museums, and the arts sector during event organization, with some even engaged in poetry research. Multiple studies have highlighted poetry’s impact on both supply and demand sides of China’s tourism industry. Most of these studies suggest that tourists tend to visit places associated with poetry [44–49]. Furthermore, since Chinese tourists generally have a strong familiarity with poetry, it is often incorporated into tour site narrations.Future efforts should focus on identifying the interests of this demographic and designing more interactive, engaging online and offline activities, such as ancient poetry word-filling competitions and poetry creation sharing sessions. This will help solidify the foundation of poetry enthusiasts and foster a rich cultural tourism atmosphere.

### Optimizing personal constraints to enhance participation

Among personal constraints, factors such as “insufficient societal guidance on appreciating ancient poetry music” and “limited time for attending ancient poetry music concerts or lectures” are significantly correlated with individuals’ engagement in ancient poetry music activities. This may stem from the scarcity of relevant music events and their monotonous formats, which limit their societal impact. Limited hosting at societal and university levels restricts the dissemination of ancient poetry music, affecting public recognition of poetry culture. The lack of activities also hinders talent cultivation in poetry music, lacking practical platforms and performance opportunities, leading to a vicious cycle. To improve this, more poetry music events should be organized, content formats enriched, and dissemination channels broadened.

For instance, cultural events can be organized to strengthen the dissemination and resonance of ancient poetry, leveraging government media for expanded publicity and increased attention. Integrating cultural and tourism resources to develop ancient poetry-themed travel routes can create immersive cultural experiences, enhancing visitors’ cultural enrichment. By harnessing community effects to broaden the reach of poetic art, a robust transmission network can be established. This will revitalize poetic art in modern society, fostering a virtuous cycle of cultural inheritance.

## Conclusions and research limitations

### Conclusions

This study introduces the theory of leisure constraints and conducts an empirical analysis to systematically examine the categories and structure of restrictions in ancient poetry experiences.It also analyzes the impact of demographic and sociological characteristics on restrictions in poetry landscape experiences. Findings reveal that both personal and structural constraints significantly impact participation and satisfaction levels. Further research indicates that specific measures to optimize these constraints include: offering customized musical experiences to heighten audience engagement; enhancing interactive features; incorporating audience feedback to continuously refine creative and performance plans; and fostering an atmosphere of active participation. These integrated strategies not only mitigate the negative effects of personal and structural constraints but also substantially boost user enthusiasm and satisfaction. Practice demonstrates that integrating personalized strategies with system optimization is the key pathway to enhancing user experience. Additionally, the study finds that strengthening recipients’ sense of belonging and offering diverse interactive opportunities effectively promotes communication and mutual assistance. This further stimulates personal constraints, forming a virtuous cycle of interaction that significantly elevates the overall experiential impact.

### Research limitations and future prospects

A limitation of this study is its sampling strategy. The sample was from specific concert attendees, potentially unrepresentative of the public. Also, self – selection bias among interested audiences may overestimate variable impacts. Future research should use more diverse sampling (e.g., non – audience at community centers or online) to improve findings’ generalizability. Moreover, the cross – sectional nature and lack of a control group make it hard to establish causal relationships. For example, the correlation between ‘immersive experience’ and ‘cultural identity’ doesn’t mean causation. Future studies could use experimental designs, comparing pre – and post – tests between a control (e.g., standard lecture participants) and an experimental group (poetry music activity participants) to test causal effects.

A notable methodological limitation of this study involves the operationalization of the ‘Participation’ construct. The measurement items assessed general attendance at classical and traditional music concerts (e.g., average number of classical concerts attended per year) rather than strictly ‘ancient poetry music concerts.’ Because dedicated ancient poetry concerts (such as Wei’s Music Score performances) represent an extremely rare and highly specialized niche, measuring specific attendance would have likely resulted in a severe statistical floor effect with near-zero variance. Consequently, general classical music participation was utilized as a necessary behavioral proxy. While this approach was essential to ensure sufficient data variance for structural equation modeling.

The Future Center will integrate ancient poetry with modern life, develop communication channels, collaborate with cultural tourism partners for exploration and activities, use digital platforms for education, combine VR and AR for immersive experiences, and expand globally via cross – industry collaborations to enhance Chinese culture’s international status. Through modern technology and social media, we’ll broaden communication, enhance interactivity, and create integrated online – offline experiences. Future plans include staging musical theater to build “Ming Music” and “Li Bai” brands, support Anhui’s culture, promote Chinese stories, and guide youth to appreciate ancient poetry and music, fostering values and cultural confidence. From a historical, cultural, and contemporary perspective, we’ll endow ancient poetry with resonance, horizons, and relevance. This aims to revitalize ancient Chinese poetry in the new era, making it popular in China and globally understood, and establishing it as an Eastern classic in art history.

## Supporting information

S1 File Original data english. (XLSX)

## References

[1] Yang S. Songs and poetry of emperor Wu of Han Dynasty and the music system of Han Yuefu. Fudan J (Soc Sci Ed). 2024;66(04):88–97.

[2] Hao Q. Significant innovative conclusions in general secretary Xi Jinping’s important discourse on the protection and inheritance of cultural heritage. China’s Intangible Cult Herit. 2025;(02):6–13.

[3] Zhang Z, Xie P. A new probe into communication path of Hakka folk songs in perspective of third creation. J Longyan Univ. 2021;39(04):36–42.

[4] Xie P, Zhang Z. Modern performance of ancient poetry and prose with tablatures from perspective of cultural inheritance: a case study of Jianghuai tide and Qingge concert. J Longyan Univ. 2025;43(01):43–9. doi: 10.16813/j.cnki.cn35-1286/g4.2021.04.006

[5] Jackson EL. Will research on leisure constraints still be relevant in the twenty-first century? J Leis Res. 2000;32(1):62–8. doi: 10.1080/00222216.2000.11949887

[6] Nadirova A, Jackson EL. Alternative criterion variables against which to assess the impacts of constraints to leisure. J Leis Res. 2000;32(4):396–405. doi: 10.1080/00222216.2000.11949923

[7] Crawford DW, Jackson EL, Godbey G. A hierarchical model of leisure constraints. Leis Sci. 1991;13(4):309–20. doi: 10.1080/01490409109513147

[8] Daniels MJ, Drogin Rodgers EB, Wiggins BP. “Travel Tales”: an interpretive analysis of constraints and negotiations to pleasure travel as experienced by persons with physical disabilities. Tour Manage. 2005;26(6):919–30. doi: 10.1016/j.tourman.2004.06.010

[9] Nyaupane GP, Andereck KL. Understanding travel constraints: application and extension of a leisure constraints model. J Travel Res. 2007;46(4):433–9. doi: 10.1177/0047287507308325

[10。] Tian S, Crompton JL, Witt PA. Integrating constraints and benefits to identify responsive target markets for museum attractions. J Travel Res. 1996;35(2):34–45. doi: 10.1177/004728759603500207

[11] Funk DC, Alexandris K, Ping Y. To go or stay home and watch: exploring the balance between motives and perceived constraints for major events: a case study of the 2008 Beijing Olympic Games. J Tour Res. 2008;11(1):41–53. doi: 10.1002/jtr.682

[12] Kim N-S, Chalip L. Why travel to the FIFA World Cup? Effects of motives, background, interest, and constraints. Tour Manage. 2004;25(6):695–707. doi: 10.1016/j.tourman.2003.08.011

[13] Tao W, Zhang S. Local residents’ sporting-event-related recreational behaviors: A case study on MGP on the basis of leisure constraints theory. Tour Tribune. 2016;31(06):50–9.

[14] Zhou W, Tian H, Qiu Y. Qualitative research on serious leisure constraints negotiation strategies of marathon runners. J Wuhan Inst Phys Educ. 2017;51(11):80–4. doi: 10.15930/j.cnki.wtxb.2017.11.013

[15] Gilbert D, Hudson S. Tourism demand constraints: a skiing participation. Ann Tour Res. 2000;27(4):906–25.

[16] Zhang HL, Zhang J. Research on the limiting factors of traditional Chinese cultural landscape experience: a case study of calligraphy landscapes. Tour Tribune. 2012;27(7):28–34.

[17] Guo YJ, He YY, Luo QJ. From passive absence to active participation: A study on leisure constraint negotiation and activity benefits of underprivileged workers’ engagement in artistic activities. Tour Tribune. 2023;38(5):137–50.

[18] Zhou LJ, Chen XY, Ren ZB, Xu MZ, Feng ZY. Construction of a three-order model for the Chinese marathon participation motivation scale. J Tianjin Univ Sport. 2020;35(4):410–4.

[19] Jin QY. An exploration of the relationship between leisure constraints and participation in leisure sports among urban residents in ethnic minority areas of Yanbian. J Nanjing Sport Inst (Soc Sci Ed). 2014;28(4):65–72, 98.

[20] Xie T, Lu N, Zhang N. The relationship between body anxiety and physical exercise in female college students: the mediating role of leisure restrictions. J Shangrao Normal Univ. 2023;43(06):86–95.

[21] Zhou W, Qiu Y, Tian H, Xu J. Women runners in China: constraints negotiation process of serious leisure. Int J Environ Res Public Health. 2021;19(1):214. doi: 10.3390/ijerph19010214 35010472 PMC8750384

[22] Canally C, Timothy DJ. Perceived constraints to travel across the US‐Mexico border among American university students. J Tour Res. 2007;9(6):423–37. doi: 10.1002/jtr.614

[23] Wan T, Zhou Y, Li S, Zhu H. Research on college students’ travelling limits and the strategy from the the constraints theory. J Dalian Univ. 2023;44(06):89–99.

[24] Chen S-F, Lou S-J, Ma S-M. Role of positive emotions in the constraint process: the case of Taiwanese college students. Leis Stud. 2018;37(5):574–88. doi: 10.1080/02614367.2018.1499798

[25] Fleischer A, Pizam A. Tourism constraints among Israeli seniors. Ann Tour Res. 2002;29(1):106–23. doi: 10.1016/s0160-7383(01)00026-3

[26] Wu HY, Lin HQ. Analysis of built environment factors affecting the participation of middle-aged and elderly people in recreational physical activities in Guangzhou City. Sports Vision. 2024;(16):23–5.

[27] Darcy S. Inherent complexity: disability, accessible tourism and accommodation information preferences. Tour Manage. 2010;31(6):816–26. doi: 10.1016/j.tourman.2009.08.010

[28] Chick G, Roberts JM. Leisure and antileisure in game play. Leisure Sci. 1989;11(2):73–84. doi: 10.1080/01490408909512208

[29] Churchill Jr GA. A paradigm for developing better measures of marketing constructs. J Mark Res. 1979;16(1):64. doi: 10.2307/3150876

[30] Hung K, Petrick JF. Developing a measurement scale for constraints to cruising. Ann Tour Res. 2010;37(1):206–28. doi: 10.1016/j.annals.2009.09.002

[31] Yu X, Xu H. Ancient poetry in contemporary Chinese tourism. Tour Manage. 2016;54:393–403. doi: 10.1016/j.tourman.2015.12.007

[32] John L, Tak-sum W. Glimpses of ancient China from classical Chinese poems. In: Proceedings of COLING 2012. 2012. pp. 621–32.

[33] Walker GJ, Halpenny E, Spiers A, Deng J. A prospective panel study of Chinese-Canadian immigrants’ leisure participation and leisure satisfaction. Leis Sci. 2011;33(5):349–65. doi: 10.1080/01490400.2011.606776

[34] Barclay D, Higgins C, Thompson R. The partial least squares (PLS) approach to causal modeling: personal computer adoption and use as an illustration. Technol Stud. 1995;2(2):285–309.

[35] Hair JF, Ringle CM, Sarstedt M. PLS-SEM: Indeed a Silver Bullet. J Mark Theory Pract. 2011;19(2):139–52. doi: 10.2753/mtp1069-6679190202

[36] Sarstedt M, Ringle CM, Hair JF. Partial Least Squares Structural Equation Modeling. In: Homburg C, Klarmann M, Vomberg A, editors. Handbook of Market Research. Cham: Springer International Publishing; 2021. pp. 587–632. doi: 10.1007/978-3-319-57413-4_15

[37] Ringle C, Wende S, Becker J. SmartPLS4. Oststeinbek: SmartPLS GmbH; 2022.

[38] Ringle J, C M, Becker S. SmartPLS 4. Oststeinbek: SmartPLS GmbH; 2022.

[39] Hair JF, Sarstedt M, Ringle CM. Rethinking some of the rethinking of partial least squares. Eur J Mark. 2019;53(4):566–84. doi: 10.1108/ejm-10-2018-0665

[40] Cohen J. Statistical power analysis for the behavioral sciences. 2nd ed. Routledge; 1988. doi: 10.4324/9780203771587

[41] Zhang H, Zhang J, Cheng S, Lu S, Shi C. Role of constraints in Chinese calligraphic landscape experience: An extension of a leisure constraints model. Tour Manage. 2012;33(6):1398–407. doi: 10.1016/j.tourman.2012.01.001

[42] Egan CH. A critical study of the origins of ‘Chüeh-chǔ’ poetry. Asia Major. 1993;6:83–125.

[43] Small C. Musicking. Wesleyan University Press; 1998.

[44] Li FMS. Chinese common knowledge, tourism, and natural landscape: Gazing on “Bie you tian di” e “an altogether different world”. Australia: Murdoch University; 2005.

[45] Packer J, Ballantyne R, Hughes K. Chinese and Australian tourists’ attitudes to nature, animals and environmental issues: Implications for the design of nature-based tourism experiences. Tour Manage. 2014;44:101–7. doi: 10.1016/j.tourman.2014.02.013

[46] Peterson YY. The Chinese landscape as a tourist attraction: images and reality. In: Lew AA, Yu L, editors. Tourism in China. Geographical, political and economic perspectives. Boulder: Westview Press; 1995. pp. 141–54.

[47] Sofield THB, Li FMS. Tourism development and cultural policies in China. Ann Tour Res. 1998;25(2):362–92. doi: 10.1016/s0160-7383(97)00092-3

[48] Xu H, Cui Q, Ballantyne R, Packer J. Effective environmental interpretation at Chinese natural attractions: the need for an aesthetic approach. J Sustain Tour. 2013;21(1):117–33. doi: 10.1080/09669582.2012.681787

[49] Xu H, Cui Q, Sofield T, Li FMS. Attaining harmony: understanding the relationship between ecotourism and protected areas in China. J Sustain Tour. 2014;22(8):1131–50. doi: 10.1080/09669582.2014.902064

