# Supplementary material for: A study of the limitations of musical experience in Ancient Chinese Poetry – The case of the creation concert of Wei’s Music Score
Source: PLoS One. 2026 Jun 11;21(6):e0346421. doi: 10.1371/journal.pone.0346421 (PMC13258017; doi:10.1371/journal.pone.0346421)
Supplement: Attachment — Submitted filename: PONE-D-25-36046.pdf [file pone.0346421.s002.pdf]

PLOS ONE

A Study of the Limitations of Musical Experience in Ancient Chinese Poetry—The Case of the Creation Concert of Wei's Music Score

--Manuscript Draft--

**Manuscript Number:**	PONE-D-25-36046
**Article Type:**	Research Article
**Full Title:**	A Study of the Limitations of Musical Experience in Ancient Chinese Poetry—The Case of the Creation Concert of Wei's Music Score
**Short Title:**	Chinese Ancient Poetry; Wei's Music Score; Leisure Restricted Class Modeling
**Corresponding Author:**	Panjie Xie, Ph.D.<br>Anhui University School of Arts<br>Hefei, CHINA
**Keywords:**	Chinese Ancient Poetry; Wei's Music Score; Leisure Restricted Class Modeling
**Abstract:**	Chinese Ancient Poetry with deep artistic value and literary value proudly in the forest of Chinese vocal works, in the new era vigorously carry forward the Chinese outstanding traditional culture background ushered in the revival of interpretation at home and abroad. At the present stage, there are communication problems such as field environment constraints, historical understanding limitations, etc. The experiential limitations of the ancient spectrum poetry will lead to the loss of the aesthetic education function of the Chinese Ancient Poetry, and there are constraints on the recipient's cultural identity, cultural self-confidence, and cultural inheritance. This study synthesizes the conceptual framework of constraints in leisure and tourism research through literature combing method, and conducts a systematic research by combing with the concerts of ancient sheet music poetry to form the constraints scale of ancient sheet music poetry experience, which is used to investigate and analyze the statistical results of the survey and analysis of the results of the recipients of three concerts of the creation of “Wei's Music Score” through the PLS-SEM method, in order to study the problem of constraints on the experience of ancient sheet music poetry. The results show that there is a three-factor structure of personal constraints, interpersonal constraints, and structural constraints in the experience of ancient poems, confirming the conclusion that there are “preference -> participation, personal constraints -> preferences, and structural constraints -> participation” in the experience of ancient poems. The study expands the application of the Leisure Restricted Class Model to traditional music, and the exploration of the restricted factors for the experience of ancient poems will help to study the restricted factors of music in different activity scenes, which is beneficial to the inheritance and promotion of the excellent traditional Chinese culture, enhances the public's cognition of and interest in the ancient poems, and provides an empirical basis for the construction of cultural self-confidence.
**Order of Authors:**	Panjie Xie, Ph.D.
	Zushun Zhang
	Jinsong Wang
**Additional Information:**	
**Question**	**Response**
**Financial Disclosure**<br><br>Enter a financial disclosure statement that describes the sources of funding for the work included in this submission. Review the submission guidelines for detailed requirements. View published research articles from *PLOS ONE* for specific examples.	Yes

This statement is required for submission and **will appear in the published article** if the submission is accepted. Please make sure it is accurate.<br><br>**Funded studies**<br>Enter a statement with the following details:<br>• Initials of the authors who received each award<br>• Grant numbers awarded to each author<br>• The full name of each funder<br>• URL of each funder website<br>• Did the sponsors or funders play any role in the study design, data collection and analysis, decision to publish, or preparation of the manuscript?<br><br>Did you receive funding for this work?	
Please add funding details.<br>as follow-up to "**Financial Disclosure**<br><br>Enter a financial disclosure statement that describes the sources of funding for the work included in this submission. Review the submission guidelines for detailed requirements. View published research articles from *PLOS ONE* for specific examples.<br><br>This statement is required for submission and **will appear in the published article** if the submission is accepted. Please make sure it is accurate.<br><br>**Funded studies**<br>Enter a statement with the following details:<br>• Initials of the authors who received each award<br>• Grant numbers awarded to each author<br>• The full name of each funder<br>• URL of each funder website<br>• Did the sponsors or funders play any role in the study design, data collection and analysis, decision to publish, or preparation of the manuscript?	Anhui Provincial Department of Education Cultivation Program for Excellent and Top-notch Talents in Colleges and Universities (Key Project of Support Program for Excellent Young Talents in Colleges and Universities) (Number:gxyqZD2022087)；Anhui Provincial Department of Education 2023 Annual College Ancient Book Arrangement Research Project (Number:2023AHGJ04)；Anhui College of the Arts “Classroom Excellence” Project: Ancient Genealogy Poetry Creation and Performance Planning (Number:2025xjzykt11).

Did you receive funding for this work?"	
Please select the country of your main research funder (please select carefully as in some cases this is used in fee calculation).<br>as follow-up to "**Financial Disclosure**<br><br>Enter a financial disclosure statement that describes the sources of funding for the work included in this submission. Review the submission guidelines for detailed requirements. View published research articles from *PLOS ONE* for specific examples.<br><br>This statement is required for submission and **will appear in the published article** if the submission is accepted. Please make sure it is accurate.<br><br>**Funded studies**<br>Enter a statement with the following details:<br>• Initials of the authors who received each award<br>• Grant numbers awarded to each author<br>• The full name of each funder<br>• URL of each funder website<br>• Did the sponsors or funders play any role in the study design, data collection and analysis, decision to publish, or preparation of the manuscript?<br><br>Did you receive funding for this work?"	CHINA - CN
**Competing Interests**<br><br>Use the instructions below to enter a competing interest statement for this submission. On behalf of all authors, disclose any competing interests that could be perceived to bias this work—acknowledging all financial support and any other relevant financial or non-financial competing interests.<br><br>This statement is required for submission	The authors have declared that no competing interests exist.

and **will appear in the published article** if the submission is accepted. Please make sure it is accurate and that any funding sources listed in your Funding Information later in the submission form are also declared in your Financial Disclosure statement.<br><br>View published research articles from *PLOS ONE* for specific examples.<br><br>**NO authors have competing interests**<br><br>Enter: *The authors have declared that no competing interests exist.*<br><br>**Authors with competing interests**<br><br>Enter competing interest details beginning with this statement:<br><br>*I have read the journal's policy and the authors of this manuscript have the following competing interests: [insert competing interests here]*<br><br>* typeset	
**Ethics Statement**<br><br>Enter an ethics statement for this submission. This statement is required if the study involved:<br><br>• Human participants<br>• Human specimens or tissue<br>• Vertebrate animals or cephalopods<br>• Vertebrate embryos or tissues<br>• Field research<br><br>Write "N/A" if the submission does not require an ethics statement.<br><br>General guidance is provided below. Consult the submission guidelines for detailed instructions. **Make sure that all information entered here is included in the Methods section of the manuscript.**	Data is collected anonymously

**Format for specific study types**<br><br>**Human Subject Research (involving human participants and/or tissue)**<br>• Give the name of the institutional review board or ethics committee that approved the study<br>• Include the approval number and/or a statement indicating approval of this research<br>• Indicate the form of consent obtained (written/oral) or the reason that consent was not obtained (e.g. the data were analyzed anonymously)<br><br>**Animal Research (involving vertebrate animals, embryos or tissues)**<br>• Provide the name of the Institutional Animal Care and Use Committee (IACUC) or other relevant ethics board that reviewed the study protocol, and indicate whether they approved this research or granted a formal waiver of ethical approval<br>• Include an approval number if one was obtained<br>• If the study involved *non-human primates*, add *additional details* about animal welfare and steps taken to ameliorate suffering<br>• If anesthesia, euthanasia, or any kind of animal sacrifice is part of the study, include briefly which substances and/or methods were applied<br><br>**Field Research**<br><br>Include the following details if this study involves the collection of plant, animal, or other materials from a natural setting:<br>• Field permit number<br>• Name of the institution or relevant body that granted permission	
**Data Availability**<br><br>Authors are required to make all data underlying the findings described fully available, without restriction, and from the time of publication. PLOS allows rare exceptions to address legal and ethical concerns. See the PLOS Data Policy and FAQ for detailed information.	Yes - all data are fully available without restriction

A Data Availability Statement describing where the data can be found is required at submission. Your answers to this question constitute the Data Availability Statement and **will be published in the article**, if accepted.<br><br>**Important:** Stating 'data available on request from the author' is not sufficient. If your data are only available upon request, select 'No' for the first question and explain your exceptional situation in the text box.<br><br>Do the authors confirm that all data underlying the findings described in their manuscript are fully available without restriction?	
**Describe where the data may be found in full sentences. If you are copying our sample text, replace any instances of XXX with the appropriate details.**<br><br>• If the data are **held or will be held in a public repository**, include URLs, accession numbers or DOIs. If this information will only be available after acceptance, indicate this by ticking the box below. For example: *All XXX files are available from the XXX database (accession number(s) XXX, XXX.*).<br>• If the data are all contained **within the manuscript and/or Supporting Information files**, enter the following: *All relevant data are within the manuscript and its Supporting Information files.*<br>• If neither of these applies but you are able to provide **details of access elsewhere**, with or without limitations, please do so. For example:<br><br>*Data cannot be shared publicly because of [XXX]. Data are available from the XXX Institutional Data Access / Ethics Committee (contact via XXX) for researchers who meet the criteria for access to confidential data.*<br><br>*The data underlying the results presented in the study are available from (include the name of the third party*	All relevant data are within the manuscript and its Supporting Information files.

and contact information or URL).<br>• This text is appropriate if the data are owned by a third party and authors do not have permission to share the data.<br><br>* typeset	
Additional data availability information:	

Click here to access/download;Manuscript;manuscript.docx

A Study of the Limitations of Musical Experience in Ancient Chinese Poetry—The Case of the Creation Concert of Wei's Music Score

Zushun Zhang[1]，Panjie Xie[1*]，Jinsong Wang[2]

1.Anhui University of Arts, Hefei, Anhui 230000

2. School of Design, Jiangnan University, Wuxi 214122, China

* Correspondence: xiepanjie@ahua.ah.cn

Abstract： Chinese Ancient Poetry with deep artistic value and literary value proudly in the forest of Chinese vocal works, in the new era vigorously carry forward the Chinese outstanding traditional culture background ushered in the revival of interpretation at home and abroad. At the present stage, there are communication problems such as field environment constraints, historical understanding limitations, etc. The experiential limitations of the ancient spectrum poetry will lead to the loss of the aesthetic education function of the Chinese Ancient Poetry, and there are constraints on the recipient's cultural identity, cultural self-confidence, and cultural inheritance. This study synthesizes the conceptual framework of constraints in leisure and tourism research through literature combing method, and conducts a systematic research by combing with the concerts of ancient sheet music poetry to form the constraints scale of ancient sheet music poetry experience, which is used to investigate and analyze the statistical results of the survey and analysis of the results of the recipients of three concerts of the creation of “Wei's Music Score” through the PLS-SEM method, in order to study the problem of constraints on the experience of ancient sheet music poetry. The results show that there is a three-factor structure of personal constraints, interpersonal constraints, and structural constraints in the experience of ancient poems, confirming the conclusion that there are “preference -> participation, personal constraints -> preferences, and structural constraints -> participation” in the experience of ancient poems. The study expands the application of the Leisure Restricted Class Model to traditional music, and the exploration of the restricted factors for the experience of ancient poems will help to study the restricted factors of music in different activity scenes, which is beneficial to the inheritance and promotion of the excellent traditional Chinese culture, enhances the public's cognition of and interest in the ancient poems, and provides an empirical basis for the construction of cultural self-confidence.

Keywords： Chinese Ancient Poetry; Wei's Music Score; Leisure Restricted Class Modeling

1. Introduction

Chinese Ancient Poetry is a splendid piece of traditional culture, a high degree of fusion of literature and music, and a concentrated manifestation of the noble sentiments of our ancestors. Ancient poems and lyrics are music carriers that cover classic poems, lyrics and compositions, including poems, lyrics and compositions, recorded in ancient Chinese notation and guqin notation (vocal score, gong-tone score, common character score, Gongshi score, Gongshi score, etc.) (Yang Sai, 2024.). In his speech at UNESCO headquarters, President Xi Jinping pointed out, “Let the cultural relics collected in museums, the heritage displayed on the vast land, and the words written in ancient books come to life, so that Chinese civilization, together with the rich and colorful civilizations created by people from all over the world, can provide correct spiritual guidance and strong spiritual impetus for mankind.” (Hao Qingjun, 2025) The performance of ancient poems through the dialogue between poetry and history to make the words have a different vitality, a successful concert of ancient poems presents the details of the performance is not designed out of thin air, nor is it independent of the work and the overall performance of the incidental behavior, but has a musical purpose and aesthetic poetic presentation of the music.

In the process of creating and performing ancient poems in the new era, there is the problem of “difficult to understand, difficult to appreciate, difficult to sing and difficult to disseminate” waiting to be solved. At present, the research direction of the academic circle mostly focuses on the collection of song sheets and the cultivation of singers, paying more attention to “one degree of creation” and “two degrees of creation”, and ignoring “three degrees of creation” (Zhang & Xie, 2021), which makes it difficult to realize the large-scale dissemination of the poems with ancient scores. The research idea centered on “third-degree creation” is to reprocess and innovate the ancient poems through modern technology and creative methods, so as to make them more in line with the aesthetic preferences of contemporary audiences. It is not only necessary to deeply explore the deep cultural heritage of ancient poems, but also need to integrate multiple elements of modern artistic expression, giving ancient poems a different artistic charm. Therefore, exploring the ways of creating and disseminating ancient poems in the new era is of far-reaching significance for the inheritance and promotion of the excellent traditional Chinese culture.

In this paper, through three original “Wei's Music Score” ancient music and poetry concerts, combined with the Partial Least Squares Structural Equation Modeling (PLS-SEM) analysis method,

we aim to provide new perspectives and ideas for researchers in this field, and explore the effective inheritance and promotion of traditional poetry and culture through the understanding of "three degrees of creativity" perceptions, participation and satisfaction. By understanding the perception, participation and satisfaction of "three degrees of creativity", we will explore innovative solutions to effectively pass on and promote traditional poetry and culture, stimulate the public's interest and empathy for traditional culture, and enhance the domestic and international influence of Chinese traditional culture.

2. Literature review

At present, domestic and foreign "leisure restrictions" research from the research surface across to the research mechanism and the diversity of research data, the research subject and the research content of a wide range, and emphasize the combination of quantitative and qualitative, theoretical and empirical methods. Early studies involved conceptual reviews (Jackson, E.L., 2000; Nadirova A& Jackson E L, 2000), studies of constraint hierarchies (intrinsic personal constraints, interpersonal constraints, and structural constraints) by researchers such as Crawford D W, Jackson E L, and others; and studies of constraint hierarchies (intrinsic personal constraints, interpersonal constraints, and structural constraints) by Kay et al, Scott; Shaw et al, and Jackson et al. Kay et al, Scott; Shaw et al and Jackson et al argued that "negotiation" can address the constraints of hierarchical relationships and overcome the limitations of intrinsic personal constraints; Jackson E L et al categorized negotiation strategies into cognitive (adjusting expectations, changing interests, etc.) and behavioral (skill learning, time management, etc.) strategies, Hubbard J and Mannell R C successfully combined conceptual analysis with theoretical reasoning to categorize the negotiation process into 4 models, which pointed out the direction for in-depth research on leisure constraints.Walker G J and Virden R J proposed a revised model of leisure constraints negotiation by combining the characteristics of outdoor recreation activities.Loucks-Atkinson et al. used social psychological theories to construct or revise the leisure constraints negotiation model, predicting people's participation in or failure to participate in outdoor recreation activities. negotiation model to predict people's participation or non-participation in leisure behavior.

Although restriction theory was proposed in the 1960s and 1970s, it is only recently that leisure restriction theory has been introduced into tourism research. Researchers have addressed nature tourism (Daniels et al., 2005; Gilbert& Hudson, 2000; Nyaupane& Andereck, 2008), museum

tourism (Jun et al., 2008; Tian et al., 1996), festival tourism (Funk et al., 1996; Kim& Chalip, 2004), sports tourism (Funk et al., 2004; Kim& Chalip, 2004; Kim& Chalip, 2004), and tourism in general. Kim&Chalip,2004) sports tourism (Tao et al.,2017;Zhou et al.,2017), cruise tourism (Sun et al.,2019) IV and many other forms of tourism have been studied for tourism constraints. In the art segment, Zhang Honglei and Zhang Jie studied the constraints of traditional Chinese cultural landscape experience (calligraphy landscape); Guo Yunjiao et al. studied the negotiation of leisure constraints and the benefits of activities for the bottom laborers' participation in art activities; in the sports segment, Zhou Liangjun et al. adopted the “motivation-constraints-negotiation” in the study of marathon participation behavior in-depth discussion; Jin Qingyun focuses on the exploration of the relationship between leisure restrictions and leisure sports participation of urban residents in Yanbian minority areas. In the process of collecting a large amount of literature, it was found that most of the research subjects were female (Zhou, W et al., 2021; Xie, T et al., 2023). Secondly there are few studies on adolescents (Agans et al.,2023;Chen et al,2018;Wan T.et al,2023) and the elderly (Hung& Petrick,2010;Wu& Lin,2024). In addition to this, research on the restrictions of leisure subjects also involves the restrictions of disabled people (Darcy S, 2009), children's restrictions (Chick & Roberts, 1989) case studies, most of the journals focusing on the study of leisure categories focus on the analysis of questionnaire data in the categories of sports and physical education, the art of painting, and the environmental arts, and the lack of a series of literature to support the study of the category of musical performances, especially the historical study of the category of ancient scores, which is of historical importance. Historical research significance of the ancient genealogy category in the field of leisure restrictions lack of case illustrations. Therefore, the present study is an in-depth analysis of Muding's performance of ancient music and poetry using the theory of leisure constraints, aiming to provide innovative insights into the cognitive attitudes of experiencers towards ancient music and poetry, and then effectively enhance their satisfaction and participation. According to the definition of leisure constraints, the constraints on the experience of Moutain poetry can be defined as factors that inhibit the continued experience of Moutain poetry, result in the inability to participate in Moutain poetry activities, maintain or increase the frequency of participation, and result in a negative impact on the quality of the experience of Moutain poetry.

Based on the literature on leisure constraints and on the characteristics of Chinese Ancient Poetry perception, we developed the Chinese Ancient Poetry Experience Constraints Scale. Using

this scale, we conducted an empirical study to examine the presence of hierarchical structure and constraint negotiation in the context of Chinese Ancient Poetry perception. The effects of constraints on preference, participation, and satisfaction were also investigated. By integrating the theory of casual constraint negotiation (Crawford et al., 1991) and its revisions in experiential contexts (Gilbert and Hudson, 2000, Hung and Petrick, 2010), this paper proposes that there are some relationships between the constraint dimensions and ancient genealogical poems' preference, participation, and satisfaction are related in a number of ways （Figure 1）.

1.1: Intrapersonal constraints have a negative effect on experiencer preferences.

1.2: Interpersonal constraints have a negative effect on experiencer participation.

1.3: Interpersonal constraints have a negative effect on experiencer satisfaction.

1.4: Structural constraints have a negative effect on experiencer participation.

1.5: Structural constraints have a negative effect on experiencer satisfaction.

Based on the theory of leisure constraints and Walker, Halpenny, Spiers, and Deng's (2011) research preferences may influence participation, and experiencers may feel more satisfied when actively participating in an activity. Therefore, we can propose two other hypotheses.

2.1: Preferences have a positive effect on participation.

2.2: Participation has a positive effect on experiencer satisfaction.

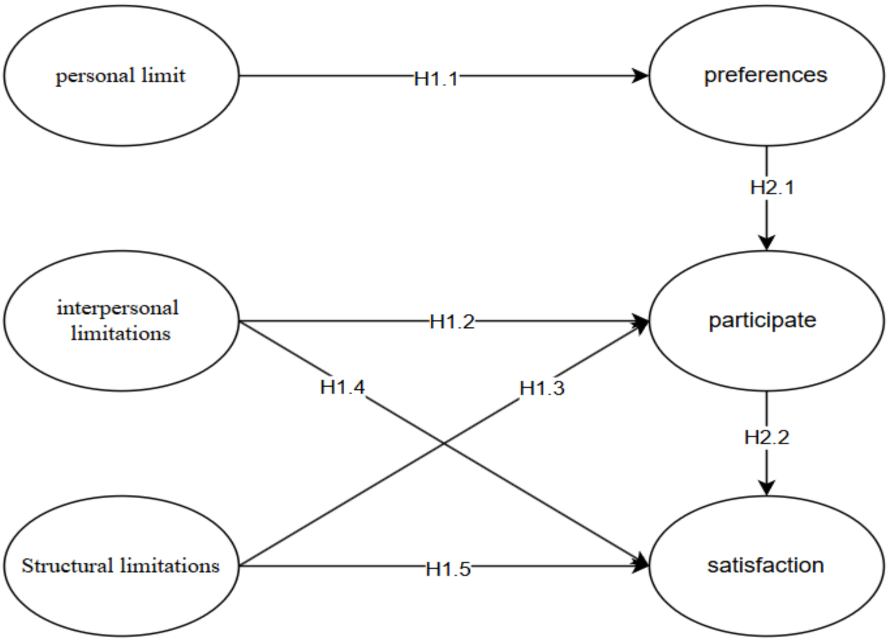

Figure 1 Restriction model of ancient genealogical poems

3. Materials and methods

3.1 Location and modalities of the study

Experiment to carry out relying on the platform of Anhui College of Arts Chinese Ancient Poetry inheritance and innovation center held three original ancient genealogy poetry concerts, one of which was sponsored by the Anhui Provincial Department of Human Resources and Social Security, the Anhui Provincial Department of Education, the Anhui College of Arts, “Wei's Music Score” Chinese Ancient Poetry inheritance training class completion activities, attracting more than 100 students from a number of provinces and the participation of 200 members of the community, the industry range involves teachers , museum members, musicians association members, social organizations and so on. The workshop invited domestic well-known experts to provide “Wei's Music Score” poetry and literature, “work of sheet music translation”, “the principles of Chinese music and literature” and other courses, before the concert set for academic lectures “Jiangnan songs and poetry and aesthetics of Jiangnan”, representative of the work of the sheet music on-site teaching and explaining the subsequent performance of the smooth implementation of laying a solid foundation. And then the concert used the guided listening intervention, the poetry literature, poets, creative background and performers to introduce, to make up for the modern appreciation of the lack of knowledge of ancient literature. Integration of scientific and technological elements, the workshop participants in advance of the collective recitation of songs corresponding to the poetry audio AI high-tech processing to generate light particles on the big screen, to bring the audience an immersive “Wei's Music Score” multimedia concert experience. After the concert, the audience with cell phones were invited to fill out electronic questionnaires by scanning the code and receive paper questionnaires without cell phones, so as to collect the viewing experience of each recipient and enrich the feedback experience（Figure 2）.

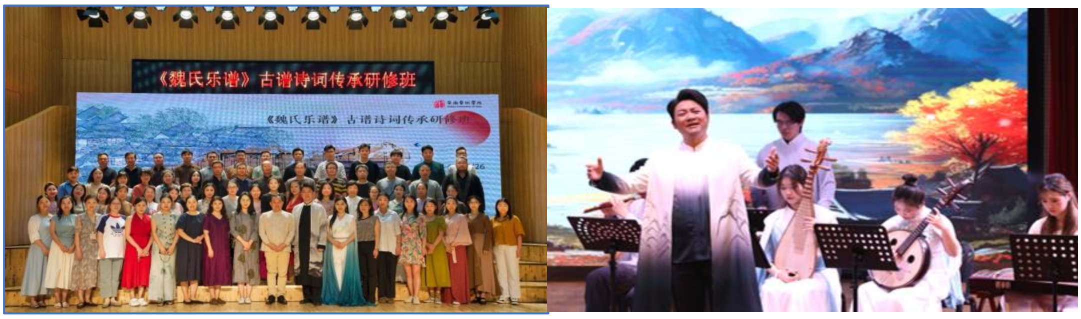

Figure 2. Pictures of the event site

The Center also co-organized two concerts with the Anhui Museum and the Hefei Musicians Association on the theme of “New Voices of Ancient Scores”, in which 300 social audiences were

selected to watch the concerts on-site by means of registration through the public number. Through the innovative arrangement of the ancient spectrum of poetry and the perfect integration of history and culture, respectively, combined with the Anhui Museum, “the world in the capital” Ming Hongwu period of fine cultural relics exhibition and 2024 cultural heritage to explain the final selection of activities will be an immersive experience, a profound excavation of ancient poetry and cultural treasures of the contemporary value of the music of the Ming Dynasty, the “real antiques”, “real antiques”, Hefei City Musicians Association jointly organized two concerts, “the new voice of the ancient spectrum”. The unique charm of “Wei's Music Score”, a “real antique” and “Ming music relic”. Before the concert, the audience can follow the guide to visit the museum “the world in the capital” Ming Hongwu period of fine cultural relics exhibition, to understand the culture of the Ming Dynasty. Through this multi-dimensional art experience, the audience can not only appreciate the beauty of the music of the ancient poems, but also to understand the historical and cultural heritage behind it, and deepen the sense of identity of traditional culture. The concert selected six groups of songs of ancient poems created by the Research Center for Inheritance and Innovation of Chinese Ancient Poetry of Anhui University of the Arts, which have a high degree of relevance to the region of Anhui, and presented the unique cultural connotation of each group of songs through the explanation of the conductor, the interpretation of the piano accompaniment, and the interpretation of the national chamber music accompaniment. A paper questionnaire and an electronic questionnaire were also distributed to ensure that the audience's opinions were fully collected, providing valuable reference for the follow-up activities. The results of the questionnaire showed that the audience highly recognized the innovative form of blending history and music, and expressed their expectation for more similar cultural experiences.

3.2 Data Acquisition and Survey Sample Analysis

The questionnaire for this study consisted of the Constraints Measurement Scale of Chinese Ancient Poetry Experience, the Preference, Participation and Satisfaction Measurement Scale, and the Demographic Characteristics and Characteristics of the Experiencers. The Constraints of Chinese Ancient Poetry Experience Measurement Scale was based on a 5-point Likert scale, with 1 indicating strongly disagree (satisfied), 2 indicating disagree (satisfied), 3 indicating fair, 4 indicating agree (satisfied), and 5 indicating strongly agree (satisfied). The paper and electronic questionnaires and interviews were conducted from August to December 2024 for the three concerts

of ancient sheet music and poetry. 553 questionnaires were distributed, 507 questionnaires were recovered, and 411 valid questionnaires were obtained after sorting out, with a recovery rate and validity rate of 91.7% and 74.3%, respectively. All procedures of this study are in accordance with institutional research guidelines and the Declaration of Helsinki 1964 and its subsequent amendments or similar ethical standards, survey answers will be collected anonymously through questionnaire star and paper questionnaires, all questionnaires were distributed with the consent of the respondents and their guardians through an informed consent form.

3.3 Sample descriptive statistics

This study collected data related to demographic characteristics. Overall, this study obtained high-quality data through offline questionnaire research, and the sample covered groups of different genders, ages, incomes and occupations, education levels, and music bases, with good representativeness and validity（Table 1）.

Table 1 Descriptive statistical analysis

Variable	Categories	Frequency	Percentage
Age	Up to 18	131	31.9
	19-24	48	11.67
	25-30	59	14.35
	31-35	47	11.43
	36-45	80	19.46
	46-55	28	6.81
	56-64	16	3.89
	65 and over	2	0.48
Sex	Male	136	33.1
	Women	275	66.9
Occupation	Office-bearer	16	4.1
	Managers of enterprises and utilities	41	10.5
	Professional/clerical technicians	91	23.3
	Service Sales Merchandisers	5	1.3
	Workers	2	0.5
	Peasants	2	0.5

	Military personnel	173	44.4
	Schoolchildren	15	3.8
	Retirees	45	11.5
educational level	Primary and below	28	7.2
	junior high school	8	2.0
	High school and secondary school	96	24.6
	three-year college	30	7.7
	undergraduate (adjective)	123	31.5
	bachelor's degree	85	21.7
	PhD	21	5.4
Music foundation	Yes	289	73.9
	No	102	26.1

The data are categorized into six ranges based on age, with 56.91% of the population aged 18-45, 31.9% under 18, and 0.48% over 65. The gender ratio is 33.1% male and 66.9% female. By industry, civil servants and managers of enterprises and institutions accounted for 12.4%, professional and technical personnel accounted for 23.3%, students accounted for 3.8%, military groups accounted for 44.4[illegible]nd retirees accounted for 11.5%. In terms of education level, 58.6% of the respondents had a bachelor's degree or above, with 27.1% having a master's degree or above. 73.9% of the respondents possessed basic music literacy, and 37.2% had attained a professional level. Music foundation is positively correlated with education level, with 81.4% of the group with liberal arts background meeting the music foundation standard, and 64.3% with science and technology. The above data show the overall trend and characteristics of the group in terms of age, gender, occupation, education and musical skills: young women with high education are the main body, the distribution of occupation shows knowledge-intensive characteristics, and there is a significant correlation between musical literacy and educational background and professional field, and this structural characteristic has important reference value for analyzing the reasons for the limitation of recreation of ancient sheet music and poetry.

4.Analysis of results

4.1 PLS-SEM analysis results

In this study, data were analyzed using Smart-PLS 4 software (J. Ringle & CM, 2022) and the reliability of the measurement instruments was systematically assessed based on the test criteria of Roldan and Cepeda (2017) and Hair et al. (2019). As shown in Figure 3 and Table 2, the factor loadings of each measurement item were higher than 0.7, and the Cronbach's alpha coefficient ($\alpha > 0.6$) together with the combined reliability ($CR > 0.7$) corroborated the internal stability of the variables. Meanwhile, the average variance extracted (AVE) of all latent variables reached the threshold of 0.5 or above, which meets the basic requirement of convergent validity. The VIF values among the variables that may be involved in formative modeling in each model are all less than 5, which can exclude the problem of covariance in model estimation. Therefore, on the whole, the scales performed well in terms of reliability and validity, providing a reliable data base for the subsequent structural modeling analysis.

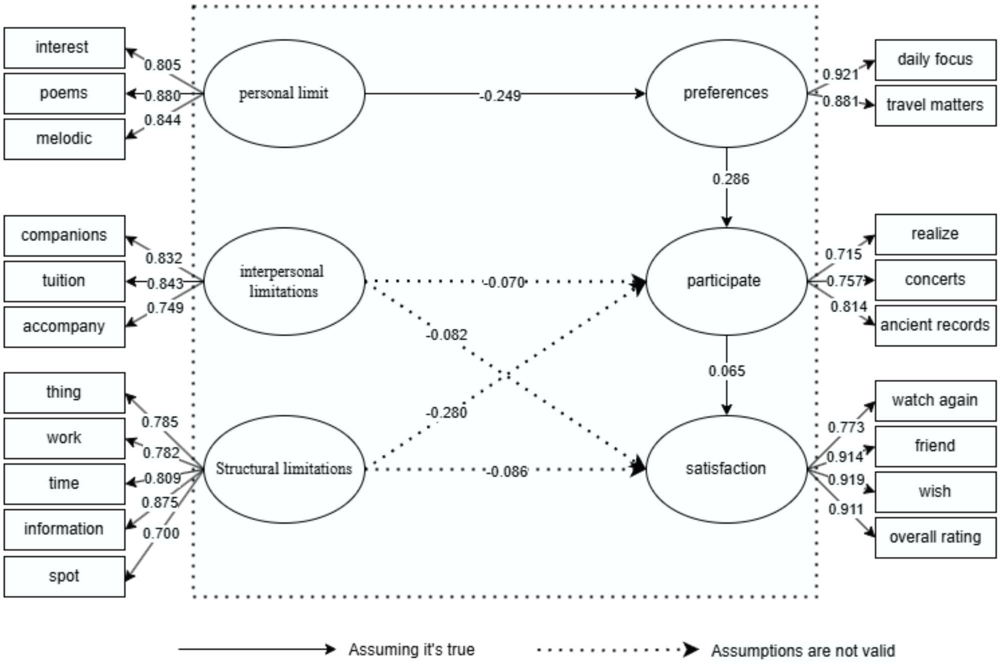

Figure 3. structural model of ancient genealogical poems

Table 2: Measures of convergent validity

Variable	Test items	Factor Load	VIF	Cronbach's a	CR	AVE
Personal limit				0.798	0.881	0.711
	You don't like to listen to concerts about old poems, you find them boring.	0.805	1.383			

	Understanding the poetic context of Chinese Ancient Poetry can be difficult for you	0.880	2.370			
	Understanding the musical melody of ancient poems can be difficult for you.	0.844	2.210			
Interpersonal limitations				0.737	0.85	0.655
	No one in your neighborhood is interested in Ancient Score Poetry Music	0.832	1.499			
	There is no one to guide you in understanding and singing the ancient poems.	0.843	1.557			
	Unaccompanied Ancient Poetry Concerts limits the number of times you can watch them.	0.749	1.372			
Structural limitations				0.851	0.893	0.628
	You are less likely to encounter things in your life that are related to the music of ancient sheet music and poetry	0.785	1.859			
	Working and living conditions limit your chances of enjoying a concert of ancient poems and lyrics	0.782	2.058			
	You don't have a lot of time to enjoy concerts or lectures on ancient music and poetry	0.809	2.182			
	There is not enough information in the society to guide you to enjoy the music of old scores and poems.	0.875	2.474			
	The distance between concerts, salons, and other events limits your participation.	0.700	1.560			
Preferences				0.771	0.896	0.812
	In your daily life, you look out for things with ancient poems (e.g. lectures, concerts, salons, etc.)	0.921	1.647			
	You will be on the lookout for things with ancient poems during your travels (e.g. museum visits, street culture, flash mobs, etc.).	0.881	1.647			
Participate				0.640	0.806	0.812
	Your level of knowledge of Chinese Ancient Poetry	0.715	1.198			
	Your average number of concerts per year	0.757	1.276			

	The average number of times you listen to classical music concerts per year	0.814	1.330			
Satisfaction				0.907	0.933	0.777
	Would you like to see a concert of ancient scores and poems about Wei's Music Score again?	0.914	3.395			
	Would you like to recommend “Wei's Music Score” Ancient Score Poetry Concert to your friends and family?	0.919	3.490			
	Would you like to see a similar concert of ancient music again in the future?	0.911	2.746			
	Your review of the Wei's Music Score Concert for the Creation of Ancient Score and Poetry	0.773	2.011			

Note: CR is the combined reliability; AVE is the variance,VIF is the covariance statistic.

According to the Fornell-Larcker criterion (Table 3), determining the discriminant validity of each construct, i.e., whether the constructs are sufficiently distinguishable from one another, involves comparing the square root of the average variance extracted (AVE) of each construct with the correlation between that construct and the other constructs. If the square root of the AVE of a construct is greater than its correlation with other constructs, then the construct satisfies discriminant validity. In summary, all constructs met the Fornell-Larcker criterion, indicating that they have good discriminant validity.

Table 3: Distinguishing Validity of Variables (Fornell-Larcker Criteria)

	Personal limit	Interpersonal limitations	Preferences	Participate	Satisfaction	Structural limitations
Personal limit	0.843					
Interpersonal limitations	0.514	0.809				
Preferences	-0.249	-0.272	0.901			
Participate	-0.194	-0.359	0.387	0.763		
Satisfaction	-0.254	-0.171	0.347	0.130	0.881	
Structural limitations	0.455	0.756	-0.292	-0.416	-0.175	0.792

The results of the path analysis indicate (see Table 5) that individuals perceived personal limitations have a significant negative effect on their preferences when participating in the Chinese Ancient Poetry experience (path coefficient = -0.249, $p < 0.001$). This result suggests that when individuals are more restricted due to time, energy, or other subjective factors, their interest in and preference for the Chinese Ancient Poetry experience will be significantly reduced. In addition, the

effect of preference on participation behavior showed a significant positive relationship (path coefficient = 0.286, $p < 0.001$), i.e., the higher the level of an individual's interest in the ancient genealogy poetry experience, the higher the likelihood that he or she will actually participate. This finding corroborates the key driving role of interest in cultural experience behavior. At the same time, it was also found that structural constraints had a similarly significant negative effect on individuals' participation behavior (path coefficient = -0.086, $p < 0.001$). That is, when external conditions (e.g., accessibility of activity resources, social support, or cultural environment) create high barriers to entry for individuals, their actual willingness to participate in the Chinese Ancient Poetry experience will be inhibited. Taken together, both personal and structural constraints have an important impact on individuals' preferences and willingness to participate, and in the future, when promoting the promotion and dissemination of the Chinese Ancient Poetry experience, attention should be paid to how to reduce the subjective and objective constraints faced by individuals in order to enhance their interest.

In partial least squares path models (SEM-PLS), the R-squared (R^2) value is an important measure of how well the model explains the variation in the dependent variable. The R^2 value ranges from 0 to 1, with higher values indicating better explanatory power of the model. Cohen (1988) defined small effect $R^2 = 0.02$, medium effect $R^2 = 0.13$ and large effect $R^2 = 0.26$) $R^2 = 0.13$ and Large effect (Large effect) $R^2 = 0.26$ were defined. As can be seen in Table 4, the R^2 for participation, satisfaction, and preference are all greater than 0.02, so a high level of explanation exists.

Table 4. Explanation of Variance

	R^2	Adjusted R^2
Participate	0.252	0.247
Satisfaction	0.038	0.031
Preferences	0.062	0.060

Table 5: Analysis of model path effect test

Effects of endogenous variables	Path coefficient	95% CI	T-value	P values	Support for assumptions
Personal limit -> Preferences	-0.249	[-0.349, -0.168]	5.11	0.000	be tenable
Interpersonal limitations -> Participate	-0.07	[-0.198, 0.079]	1.016	0.310	untenable
Interpersonal limitations -> Satisfaction	-0.082	[-0.241, 0.069]	0.99	0.323	untenable
Preferences -> Participate	0.286		6.356	0.000	

		[0.199, 0.375]			be tenable
Participate -> Satisfaction	0.065	[-0.069, 0.191]	0.948	0.343	untenable
Structural limitations -> Participate	-0.28	[-0.395, -0.152]	4.549	0.000	be tenable
Structural limitations -> Satisfaction	-0.086	[-0.237, 0.079]	1.069	0.286	untenable

4. Discussion

The above data indicate that personal and structural constraints have a significant effect on preference, while the effect of interpersonal constraints is not significant. The positive effect of preference on participation was significant, but the direct effect of participation on satisfaction was weak. This is similar to the effect of intra-personal on preference and the effect of preference on participation in the calligraphic landscape paper (Zhang, Zhang, Cheng, Lu, & Shi, 2012). This suggests that we need to focus on individual and structural level factors when enhancing participation and satisfaction. Meanwhile, the mediating role of preferences can also be ignored, and its facilitating effect on participation is particularly obvious. Further analysis shows that the negative effects of personal and structural constraints are significant, suggesting that these constraints play a key role in hindering participation and satisfaction. Optimization strategies should focus on mitigating these constraints to effectively increase overall participation and satisfaction levels. In addition, the positive effect of preferences on engagement reveals the importance of users' intrinsic motivation, which needs to be further stimulated to help overcome the constraints and enhance engagement. At the same time, structural constraints are optimized to create more favorable field scenarios for the performance of ancient genealogical poems in order to achieve both participation and satisfaction.

4.1 Addressing Personal Constraints to Enhance Preference Values

"Understanding the poetic context of ancient poems is difficult for you" has a factor loading of 0.880 in the individual limitations, indicating that the difficulty of understanding the context of ancient poems is the main factor influencing preferences. The factor loading of "It is difficult for you to understand the music melody of ancient poems" in the personal limitations is 0.844, which shows that the difficulty of understanding the music melody also significantly affects the preference. This may be due to the fact that ancient poetry is often rich in historical and cultural connotations, involves important knowledge of festivals, geography, and history (LEE & Tak-sum, 2012), consists

of structured language that deviates from everyday language use (Egan, C.H), and its poetic genre imposes unique combinatorial constraints on linguistic elements (Egan, C.H), which demands a high level of literary literacy and artistic skills are quite demanding. This can be addressed by providing a detailed concert optimization strategy to reduce comprehension difficulty, thus enhancing users' preference for ancient poetry.

4.1.1 Concert Optimization Path

The construction of field space has a significant impact on concert content and form. Bourdieu defines field as a network of objective relationships between locations. Most of the current ancient spectral performance venues are one-way output theater concert halls, where the stage and the auditorium are far away from each other, dividing the different relational networks into classes. Christopher Small, a renowned sociologist, states, "The modern concert hall is based on the assumption that musical performance is a one-way transmission system in which the composer's creative intent is communicated to the audience through the medium of the performer." (Small C, 1998) The one-way transmission model reduces the interaction between the audience and the performer, which affects the audience's ability to experience the emotion and engagement of the ancient score poetry. Optimizing the design of the venue to shorten the distance and enhance the interactivity is the key to enhance the performance. Immersive environments can stimulate emotional resonance and increase engagement and satisfaction. For example, in the performance, the performers "live" the lyrics through body language, determine the roles according to the lyrics, and show the roles vividly in front of the audience through the display of important verbs, so that the language has performativity and creativity; the performance costumes, as an easy-to-observe symbol of the performance, make up for the harsh requirements of the distance of the eye contact, through which the audience can have a better understanding of the background of the work. Through this symbol, the audience can have a more intuitive understanding of the background of the work and the characters; the guide guides the audience into the world of ancient music with vivid language, analyzes the cultural connotation behind the poems, and connects the audience and the performers like a bridge.

Through the above reshaping of the poetic mood, the receiver can reconstruct the cognition in immersion, the mind resonates with the ancient rhyme, the cultural essence can be internalized, and the emotional resonance realizes the mingling of history and modernity, and deepens the

understanding and love for the art of ancient genealogy. It inspires the audience to pay attention to lectures, concerts, salons, street culture and other things with ancient music and poetry in their daily life and travel, enhances the popularization and dissemination of the art of ancient music and music, revitalizes traditional culture in modern society, and guides the audience to become practitioners of cultural inheritance in their participation.

4.1.2 Online promotion path

We provide a rich and diverse range of online music healing resources for listeners through our self-owned public number "New Sound of Ancient Score". The public number regularly publishes knowledge, case studies and event previews to enhance public awareness and interest in music healing through ancient music and poetry. At the same time, it cooperates with museums, musicians' associations, TV stations and music broadcasters to further stimulate the public's interest in and love of traditional culture. Using interactive features such as comments and polls to collect user feedback and needs, we continuously optimize and adjust the promotional content to ensure that the healing resources provided are more relevant to the actual needs of users. Media promotion is combined with offline activities to form an online and offline linkage publicity model, further expanding the influence of the music healing of ancient poems. In addition, with the help of social media platforms, such as Weibo and Jitterbug, short videos and live broadcasts are released to show the charm of ancient poems and attract the attention of young groups. Regularly organize online concerts and poetry recitals, inviting famous artists to participate, enhancing interactivity and user stickiness. Through data analysis, we accurately locate the target audience and formulate personalized promotion strategies to ensure that the communication effect is maximized. Through multi-channel linkage, the music healing of ancient poems and lyrics is gradually integrated into the public's life, becoming a remedy for spiritual comfort. Users deepen their recognition of traditional culture through interaction and spontaneous dissemination, forming a virtuous cycle and promoting the vitality of the art of ancient music in the new era.

4.2 Using preference orientation to stimulate engagement

The group of people with the preference "In your daily life, you pay attention to things with ancient poetry (e.g., lectures, concerts, salons, etc.)" (a factor loading of 0.921) has a direct impact on their active participation in poetry events. The reason for this is that in organizing events, ancient poetry has been found to have loyal followers from universities, museums, and art fields, and some

of them are even engaged in poetry research. Several studies have now pointed out the impact of poetry on both the supply and demand sides of the Chinese tourism industry (Li, 2005; Packer, Ballantyne,&Huges,2014; Peterson, 1995; Sofield & Li, 1998; Xu,Cui,Ballantyne,& Packer, 2013; Xu,Cui, Sofield, & Li, 2014; Xu, Ding, & Packer, 2008) Most of these studies concluded that tourists tend to visit places associated with poetry (Sofield & Li, 1998). In addition, since Chinese visitors are generally aware of poetry, they also use poetry in the explanations of tourist attractions (Sofield & Li, 1998).Subsequently, it is necessary to grasp the interest points of such people and design more interactive and participatory online and offline activities, such as ancient poetry word-filling contests and poetry creation sharing sessions, so as to build a firm foundation for poetry lovers and form a strong cultural tourism atmosphere. Sun et al. in "Study on the Travel Restrictions and Behavioral Intentions of Chinese Cruise Tourists" proposed that we should differentiate between real and potential tourists, and target to stimulate the intention to travel. While maintaining the existing tourists, fully exploring potential tourists is an important way to enhance the market share of cruise tourism, increase economic income and promote the sustainable development of cruise tourism. Some scholars have suggested that Chinese poetry can be seen as embedded in the many elements that make up Chinese culture as a whole. It provides a unique window for understanding Chinese culture through its distinctive features and connections with other elements (Yu & Xu, 2015). For ancient poetry enthusiasts, exclusive experiential activities, such as ancient poetry appreciation salons and interactive workshops, can be planned to deepen the sense of participation. And facing the first-time experiencers to provide introductory lectures, simple ancient poetry teaching, lowering the threshold of participation, stimulating interest, and gradually guiding them to explore poetry literature and art in-depth, realizing the wide coverage of cultural heritage. Potential tourists are prompted to adopt a more positive negotiation strategy towards their intrinsic personal constraints, alleviating to a certain extent the inhibiting effects of travel restrictions such as time, money, family obligations, and lack of companions.

4.3 Optimize structural constraints to enhance participation

Among the structural limitations, “There is not enough information in the society to guide you to appreciate the music of ancient music and poetry” and “You don't have much time to enjoy concerts or lectures on ancient music and poetry” have higher influence factors. The reason may be that the current activities related to music with ancient scores are organized less frequently and in a

single form, which makes it difficult to form a wide social influence. The organization of activities at the social level and the university level limits the scope of the dissemination of genealogical music, and affects the public's awareness and acceptance of genealogical music. The lack of activities also leads to the difficulties in the cultivation and excavation of music talents, and the lack of sufficient practice platforms and display opportunities, which makes the inheritance and development of ancient music fall into a vicious circle. In view of this situation, it is necessary to increase the efforts of organizing activities of ancient music, enrich the content and form, and broaden the dissemination channels.

4.3.1 Increase publicity and promotion in conjunction with the government media

Jointly organize cultural lectures, exhibitions and other activities to enhance the social influence of Chinese Ancient Poetry. Through the credibility of government media, expand the coverage of publicity and attract the attention of more groups. Regularly release special reports, in-depth interpretation of the art of ancient genealogy, and enhance public awareness. Multi-party linkage to form a publicity synergy to promote the culture of ancient genealogy in the hearts of the people. Using the reward mechanism and honor certification to motivate participants to continue to invest in the art of ancient genealogy, and gradually cultivate them into loyal fans, which in turn drives more people to pay attention to and inherit this ancient art. Guide enthusiasts to form a genealogy community, exchange ideas and share resources on a regular basis, forming a close learning community. Organize online and offline workshops, invite experts to guide and enhance the professional skills of the enthusiasts; design diversified interactive links, such as online poetry solitaire and offline guzheng performance experience, to enhance the sense of participation and immersion. Through the radiation effect of the community, expand the influence of the art of ancient genealogy, attract more potential audiences, gradually build up a solid inheritance network, promote the art of ancient genealogy in the modern society, and form a benign ecosystem of cultural inheritance.

4.3.2 Strengthening in-depth integration of culture and tourism

Integrate cultural and tourism resources, launch ancient genealogy and poetry themed tourism lines, create immersive cultural experiences, and enhance tourists' sense of cultural acquisition. Combined with local characteristics, the development of ancient genealogy cultural and creative products, enrich the tourism market, promote cultural consumption, and realize the economic

benefits and cultural heritage win-win situation. Through the Chinese Ancient Poetry poetry theme tourism line, tourists can not only enjoy the beautiful scenery, but also personally experience the charm of ancient genealogy, and enhance cultural identity. At the same time, with the help of cultural and creative products, the elements of ancient genealogy are integrated into daily life, stimulating the public's love for traditional culture and forming a lasting cultural influence.

By optimizing the activity schedule and introducing flexible participation modes, such as segmented lectures and online live broadcasting, we can reduce the impact of time limitations; at the same time, we can provide diversified ticket prices and preferential packages to reduce the economic burden and attract more people to participate; we can offer parent-child genealogy courses to promote joint family learning and enhance parent-child relationships, as well as to cultivate the next generation's interest in genealogy; and we can combine with community activities to draw the distance between genealogy art and Combined with community activities, it brings the art of ancient genealogy closer to daily life, improves the convenience of participation, gradually eliminates structural limitations, and allows more people to easily come into contact with and fall in love with the culture of ancient genealogy, and gradually forms a stable community of ancient genealogy culture. Through online and offline interactions, break the geographical restrictions, broaden the social circle, and enhance the enthusiasm of participation. Enhance participation and satisfaction, realize the wide dissemination of Chinese Ancient Poetry culture and maximize user value. At the same time, innovative content, combined with modern elements, to enhance the attractiveness of ancient genealogy poetry. Strengthen community construction, promote cultural exchanges, and form a stable popularization ecology of ancient genealogy. Develop a multi-dimensional improvement program to enhance participation and satisfaction and promote the virtuous cycle of the ancient genealogy system.

5. Conclusions and future perspectives

This paper introduces the theory of leisure constraints and systematically analyzes the categories, structures, and negotiation processes of constraints in the experience of Chinese Ancient Poetry through empirical research, and also analyzes the effects of demographic and sociological characteristics on constraints in the experience of ancient genealogical landscapes. It was concluded that personal and structural constraints have a significant impact on participation and satisfaction. Further research suggests that specific measures to optimize personal and structural constraints

include providing a customized musical experience to increase the recipient's interest in attending the performance, as well as optimizing interactive features, incorporating audience feedback, and continually improving the creation of the performance program to create an atmosphere of active participation. Through these comprehensive measures, not only can the negative impact of personal and structural limitations be effectively mitigated, but also the enthusiasm and satisfaction of users can be significantly enhanced. The combination of personalized strategies and system optimization has proven to be a key path to enhancing user experience. In addition, it was found that by enhancing the sense of belonging of recipients and providing diverse interaction opportunities, communication and mutual support can be effectively promoted to further stimulate the enthusiasm for participation and form a virtuous interaction cycle, thus significantly enhancing the overall experience effect.

Inheritance and innovation of ancient poems in the new era need to focus on the audience's perception and understanding of the creation of the performance, interaction in the performance, feedback after the interaction, so that it has a constant regenerative capacity and strong attraction. Through the changes of the past and present, the essence of the performance is extracted from the traditional ideas of Chinese language habits and emotional identity, and the interpreters, creators, performers and receivers are closely related to each other, jointly presenting the beauty of the retro and the beauty of innovation. Nowadays, relying solely on the research of singing performance ideas can not maximize the use of existing resources to make the dissemination effect more far-reaching, we should use the philosophical thinking of the big historical view, the big view of the times, the Chinese Ancient Poetry forging as a carrier of traditional culture, relying on and not limited to the performance, to obtain the recognition of the masses and the dissemination of the formation of the Chinese people's common sense of nationalism, and to promote the cultural self-consciousness, cultural self-confidence.

In the future, the Center will focus on deepening the integration of Chinese Ancient Poetry with modern life, and exploring more diversified ways of dissemination; cooperating with culture and tourism to tap into regional cultural characteristics and create thematic experiential activities, so as to enhance interactivity and participation; combining with digital platforms to promote online education courses, popularize knowledge of ancient genealogy, and cultivate the interest of the younger generation; and expanding the international market through cross-border cooperation, so as to enhance the influence of Chinese culture, and realize the global dissemination of Chinese

Ancient Poetry. The globalization of ancient genealogical poems and lyrics. To rehearse a musical stage play interpretation of Li Bai's ancient score of 8, will be Anhui College of the Arts Chinese Ancient Poetry inheritance and innovation research center has completed the scientific research results of the “Wei's Music Score” work of sheet music translation and creation of vocal works through the form of musical stage play to the general public, for schools, enterprises and institutions and the community to dedicate the cultural feast. To create Anhui's “Mingle” brand of culture and tourism and “Li Bai” brand of culture and tourism, help Anhui Anhui Wanjiang culture, and contribute to the cultural strength of the province. Fully develop Li Bai's ancient poetry and music healing function, tell a good Chinese story, lead the audience, especially young people to recognize, feel, understand ancient Chinese poetry and music, set up a correct outlook on life and values, enhance cultural self-awareness and firm cultural self-confidence, and promote the development of mental health. Through modern technology and media, the program utilizes social media to broaden communication channels, enhance interactivity, and create an online and offline fusion of cultural experiences. Combined with VR and AR technology, it provides immersive viewing and deepens emotional links, and gives Chinese ancient poems a deeper historical background, a broader cultural vision, and more distinctive characteristics of the times with a larger historical view, a larger cultural view, and a larger view of the times. Chinese ancient poems will definitely be revitalized in the new era, accepted by the people of China and understood by the world, and become the magnificent oriental classics in the history of human art.

Funding Declaration: Anhui Provincial Department of Education Cultivation Program for Excellent and Top-notch Talents in Colleges and Universities (Key Project of Support Program for Excellent Young Talents in Colleges and Universities) (Number:gxyqZD2022087)；Anhui Provincial Department of Education 2023 Annual College Ancient Book Arrangement Research Project (Number:2023AHGJ04)；Anhui College of the Arts “Classroom Excellence” Project: Ancient Genealogy Poetry Creation and Performance Planning (Number:2025xjzykt11).

Declaration of Competing Interest: The authors declare that they have no known competing financial interests or personal relationships that could have appeared to influence the work reported in this paper.

Data availability: The original contributions presented in the study are included in the article/Supplementary material, further inquiries can be directed to the corresponding author.

Author contributions: ZS and PJ: conceptualization, validation, writing–review and editing, supervision, and project administration. ZS and PJ: methodology and writing draft preparation. JS: software and visualization. ZS and PJ: formal analysis. ZS and PJ: investigation. ZS and PJ: resources. JS: data curation. All authors have read and agreed to the published version of the manuscript.

Ethical approval： All questionnaires in this study were administered with the consent of the respondents and without privacy implications.

Reference

Agans, J., Son, J. S., Hanna, S., Powers, S., & Weybright, E. (2023/online first).College students’ healthy leisure:Latent profile analyses of constraints and negotiation strategies. Leisure Sciences. Impact Factor > 1 (4.00). https://doi.org/10.1080/01490400.2023.2252434

Cai, Z.-Q.(2008). How to read Chinese poetry: A guided anthology(Columbia University Press).

Chen, S.-F., Lou, S.-J., & Ma, S.-M. (2018a). Role of positive emotions in the constraint process: the case of Taiwanese college students. Leisure Studies, 1–15. https://doi.org/10.1080/02614367.2018.1499798

Chick G，Roberts J M.(1989). Leisure and antileisure in game play．Leisure Sciences，11 (2) : 73 — 84．

Cohen, J. (1988). Statistical Power Analysis for the Behavioral Sciences (2nd ed.). Routledge. doi:https://doi.org/10.4324/9780203771587

Crawford, D. W., Jackson, E. L., & Godbey, G. (1991). A hierarchical model of leisure constraints. Leisure Sciences, 13(4), 309-320.

Daniels M J， Drogin Rodgers E B，Wiggins B P．（2005）.Travel Tales: An interpretive analysis of constraints and negotiations to pleasure travel as experienced by persons with physical disabilities． Tourism Management，26 (6) : 919 —930．

Egan, C.H.(1993). A Critical Study of the Origins of’ Chüeh-chǔ’ Poetry.Asia Major 6, 83-125.

FUDAN JOURNAL(Social Sciences), 66 (04), 88-97.

Funk, D. C., Alexandris, K., & Ping, Y. (2009). To go or stay home and watch: exploring the balance between motives and perceived constraints for major events: a case study of the 2008 Beijing Olympic Games. International Journal of Tourism Research, 11(1), 41–53. https://doi.org/10.1002/jtr.682

Gilbert D，Hudson S.(2000) .Tourism demand constraints: A skiing participation． Annals of Tourism Research，27 (4) :906 — 925．

Gilbert, D., & Hudson, S. (2000). Tourism demand constraints: a skiing participation. Annals of Tourism Research,

27(4), 906-925.
Hair, J. F., Sarstedt, M., & Ringle, C. M. (2019). Rethinking some of the rethinking of partial least squares. European journal of marketing, 53(4), 566-584.
Hao Qingjun. (2025). Significant Innovative Conclusions in General Secretary Xi Jinping's Important Discourse on the Protection and Inheritance of Cultural Heritage. China's Intangible Cultural Heritage, (02), 6-13.
Hung, K., & Petrick, J. F. (2010). Developing a measurement scale for constraints to cruising. Annals of Tourism Research, 37(1), 206-228.
Hung, K., & Petrick, J. F. (2010). Developing a measurement scale for constraints to cruising. Annals of Tourism Research, 37(1), 206–228. https://doi.org/10.1016/j.annals.2009.09.002
Jackson, E.L. (2000) Will Research on Leisure Constraints Still Be Relevant in the Twenty-First Century? Journal of Leisure Research, 32, 62-68.
John,L,Tak-sum,W.（2012）.Glimpses of Ancient China from Classical Chinese Poems.Proceedings of COLING. 2012:621–632.
Kim N S，Chalip L.(2004).Why travel to the FIFA World Cup? Effects of motives，background，interest，and constraints． Tourism Management,25 (6) : 695 — 707．
Li, F. M. S. (2005). Chinese common knowledge, tourism, and natural landscape: Gazing on “Bie you tian di” e “an altogether different world”. Unpublished PhD thesis.Australia: Murdoch University.
Nadirova A，Jackson E L． (2000) Alternative criterion variables against which to assess the impacts of constraints to leisure. Journal of Leisure Research，32 (4) : 433 —439．
Nyaupane G P，Andereck K L.(2008).Understanding travel constraints:Application and extension of a leisure constraints model． Journal of Travel Research，46(4) : 433 — 439.
Packer, J., Ballantyne, R., & Huges, K. (2014). Chinese and Australian tourists' atti-tudes to nature, animals and environmental issues: implications for the design of nature-based tourism experiences. Tourism Management, 44, 101-107.
Peterson, Y. Y. (1995). The Chinese landscape as a tourist attraction: images and reality. In Alan A. Lew, & Lawrence Yu (Eds.), Tourism in China. Geographical, political and economic perspectives (pp. 141-154). Boulder: Westview Press.
Ringle, C., Wende, S., & Becker, J. (2022). SmartPLS4 (Ver-sion 4)[Computer Software]. Oststeinbek: SmartPLS GmbH. In.
Ringle, J., & CM, W. (2022). S., and Becker, “SmartPLS 4.” Oststeinbek: SmartPLS GmbH. In.
Roldan, J., & Cepeda, G. (2017). PLS-SEM. En CFP, Universidad de Sevilla, 4.
Sarstedt, M., Ringle, C. M., & Hair, J. F. (2017). Partial Least Squares Structural Equation Modeling. In C. Homburg, M. Klarmann, & A. Vomberg (Eds.), Handbook of Market Research (pp. 1-40). Cham: Springer International Publishing.
Sofield, T. H. B., & Li, F. M. S. (1998). Tourism development and cultural policies inChina. Annals of Tourism Research, 25(2), 362-392.
Sun,X, Xu.M&Hou.Y. (2019). A Study on Chinese Cruisers’ Travel Constraints and Behavioral Intentions .Tourism Science, 33 (04), 70-84. doi:10.16323/j.cnki.lykx.2019.04.005.
Tao.W & Zhang.S. (2016). Local Residents’ Sporting-Event-Related Recreational Behaviors:A Case Study on MGP on the Basis of Leisure Constraints Theory. Tourism Tribune, 31 (06), 50-59.
Tian, S., Crompton, J. L., & Witt, P. A. (1996). Integrating constraints and benefits to identify responsive target markets for museum attractions. Journal of Travel Research, 35(2), 34–45. https://doi.org/10.1177/004728759603500207
Walker, Gordon J., Halpenny, E., Spiers, A., & Deng, J. (2011). A prospective panel study of Chinese-Canadian

immigrants' leisure participation and leisure satisfaction. Leisure Sciences, 33(5), 349-365.

Wan.T, Zhou.Y, Li.S& Zhu. H (2023). Research on College Students' Travelling Limits and the Strategy from the the Constraints Theory. Journal of Dalian University , 44 (06), 89-99.

Wu Huiyu & Lin Haiqi. (2024). Analysis of Built Environment Factors Affecting the Participation of Middle-Aged and Elderly People in Recreational Physical Activities in Guangzhou City. Sports Vision, (16), 23-25.Darcy S.(2009).Inherent complexity: Disability，accessible tourism and accommodation information preferences .Tourism Management，31 (6) : 816 — 826.

Xie.T, Lu.N&Zhang.N. (2023). The Relationship between Body Anxiety and Physical Exercise in Female College Students: The Mediating Role of Leisure Restrictions. Journal of Shangrao Normal University, 43 (06), 86-95.

Xu, H., Cui, Q., Ballantyne, R., & Packer, J. (2013). Effective environmental inter-pretation at Chinese natural attractions: the need for an aesthetic approach.Journal of Sustainable Tourism, 21(1), 117-133.

Xu, H., Cui, Q., Sofield, T., & Li, F. M. S. (2014). Attaining harmony: understanding the relationship between ecotourism and protected areas in China. Journal of Sus-tainable Tourism, 22, 1131-1150.

Xu, H., Cui, Q., Sofield, T., & Li, F. M. S. (2014). Attaining harmony: understanding the relationship between ecotourism and protected areas in China. Journal of Sus-tainable Tourism, 22, 1131-1150.

Yang Sai. (2024). Songs and Poetry of Emperor Wu of Han Dynasty and the Music System of Han Yuefu

Yu,X., Xu,H,.(2016).Ancient poetry in contemporary Chinese tourism.Tourism Management,54(6):393-403.https://doi.org/10.1016/j.tourman.2015.12.007.

Zhang, H., Zhang, J., Cheng, S., Lu, S., & Shi, C. (2012). Role of constraints in Chinese calligraphic landscape experience: An extension of a leisure constraints model. Tourism Management, 33(6), 1398 – 1407. https://doi.org/10.1016/j.tourman.2012.01.001

Zhang.Z & Xie.P. (2021). A New Probe into Communication Path of Hakka Folk Songs in Perspective of Third Creation.Journal of Longyan University, 39 (04), 36-42. doi:10.16813/j.cnki.cn35-1286/g4.2021.04.006.

Zhou, W., Qiu, Y., Tian, H., & Xu, J. (2021a). Women runners in China: Constraints negotiation process of serious leisure. International Journal of Environmental Research and Public Health, 19(1), 214. https://doi.org/10.3390/ijerph19010214

Zhou.W，Tian.H& Qiu.Y. (2017). Qualitative Research on Serious Leisure Constraints Negotiation Strategies of Marathon Runners. Journal of Wuhan Institute of Physical Education, 51 (11), 80-84. doi:10.15930/j.cnki.wtxb.2017.11.013.

Click here to access/download
Supporting Information
Statement of knowledge2.docx
