# Supplementary material for: A study of the limitations of musical experience in Ancient Chinese Poetry – The case of the creation concert of Wei’s Music Score
Source: PLoS One. 2026 Jun 11;21(6):e0346421. doi: 10.1371/journal.pone.0346421 (PMC13258017; doi:10.1371/journal.pone.0346421)
Supplement: Attachment — Submitted filename: Response to Reviewers.docx [file pone.0346421.s003.docx]

Dear editor:

We sincerely appreciate the valuable comments provided by the reviewers. All modifications made in the manuscript are highlighted in blue.

Journal Requirements:

**1.Editorial comments：**1. "It appears that your ORCiD iD has not been validated in your Editorial Manager account and we are unable to proceed until that step is complete.

**Our response：We appreciate the editor's feedback and have re-verified the ORCiD.**

**2.Editorial comments：**Can you please upload an additional copy of your revised manuscript that does not contain any tracked changes or highlighting as your main article file. This will be used in the production process if your manuscript is accepted. Please amend the file type for the file showing your changes to Revised Manuscript w/tracked changes.

**Our response：**Thank you for your feedback, editor. I have reinserted the manuscript without any annotations.

**3. Editorial comments ：**Please remove your figures/ from within your manuscript file, leaving only the individual TIFF/EPS image files. These will be automatically included in the reviewer’s PDF.

**Our response：**We appreciate the editor's feedback and have removed the figures from the manuscript, adding them to the supporting files.

**4. Editorial comments**：Your ethics statement should only appear in the Methods section of your manuscript. If your ethics statement is written in any section besides the Methods, please delete it from any other section.

**Our response：**Based on the editor's valuable feedback, we have removed all ethical justification content except for the methodology section.

**5.Editorial comments：**We understand that your data availability statement currently reads as follows: "All relevant data are within the manuscript and its Supporting Information files."However, we note that portions of the file "Original data.xlsx" are in Chinese.

**Our response：**We appreciate the editor's valuable feedback and will resubmit the original data in English.

**6.Editorial comments：** Thank you for letting us know that there was consent obtained. Please provide a PLOS consent form for the people in the images in Figure 2: https://journals.plos.org/plosone/s/downloads-and-translations

**Our response：**We appreciate the editor's valuable feedback. Figure 2 contained poster information and did not depict personnel; it has been removed from the manuscript.

We would like to express our sincere gratitude once again to the reviewers and editors for their valuable suggestions. We have carefully revised the manuscript based on your feedback and hope that our research will meet the journal's standards. Should there be any further shortcomings, please do not hesitate to point them out.
