# Supplementary material for: A study of the limitations of musical experience in Ancient Chinese Poetry – The case of the creation concert of Wei’s Music Score
Source: PLoS One. 2026 Jun 11;21(6):e0346421. doi: 10.1371/journal.pone.0346421 (PMC13258017; doi:10.1371/journal.pone.0346421)
Supplement: Attachment — Submitted filename: Review Comments Manuscript ID [PONE-D-25-36046R1].docx [file pone.0346421.s004.docx]

**Manuscript ID:** [PONE-D-25-36046R1]
**Manuscript Title:** A Study of the Limitations of Musical Experience in Ancient Chinese Poetry - The Case of the Creation Concert of Wei’s Music Score

**Overall Evaluation**

This study applies leisure constraints theory to ancient poetry experience, and the topic is interesting. The research methods are appropriate, the data analysis is generally sound, and the overall structure is clear. However, the manuscript still needs improvement in methodological reporting and result interpretation. Below are specific suggestions for the final polishing stage.

**Suggestions**

**1. Sample size justification**

The authors did not explain how the sample size was determined. PLS-SEM typically follows the 10-times rule,which means the sample should exceed 10 times the largest number of paths pointing to a single construct. Please clarify whether the sample of 411 meets this requirement.

**2. Causal language**

The manuscript uses the word “influence” in several sections. Because this study relies on cross-sectional data, it can only demonstrate correlations rather than direct causality. Please replace “influence” with terms such as “predict” or “correlate with” in some expressions.

**3. Model explanatory power**

The R² values for preference (0.062) and satisfaction (0.038) are low. The authors’ explanation is reasonable, but the discussion could be expanded to acknowledge potential omitted variables, such as cultural capital or prior poetry exposure.

**4. Sentence length**

Some sentences are too long. Breaking them into shorter sentences would improve readability.

**Summary**

The study is methodologically sound and the conclusions are credible. With improvements in reporting, interpretation, and language, the manuscript will be stronger. Please make these revisions during the final polishing stage.
