# Supplementary material for: A study of the limitations of musical experience in Ancient Chinese Poetry – The case of the creation concert of Wei’s Music Score
Source: PLoS One. 2026 Jun 11;21(6):e0346421. doi: 10.1371/journal.pone.0346421 (PMC13258017; doi:10.1371/journal.pone.0346421)
Supplement: Peer review history

# Peer review: Decision Letter 0

**DOI:** 10.1371/journal.pone.0346421.r001

**Authors:** Jie Zeng

24 Sep 2025

PONE-D-25-36046

A Study of the Limitations of Musical Experience in Ancient Chinese Poetry—The Case of the Creation Concert of Wei's Music Score

PLOS ONE

Dear Dr. Xie,

Thank you for submitting your manuscript to PLOS ONE. After careful consideration, we feel that it has merit but does not fully meet PLOS ONE’s publication criteria as it currently stands. Therefore, we invite you to submit a revised version of the manuscript that addresses the points raised during the review process.

The reviewers and I acknowledge the merit of your work. However, we have decided that your manuscript requires major revisions before it can be accepted for publication. The reviewers have provided constructive feedback, primarily concerning the methodology, language, and scientific rigor of the study. I concur with their suggestions.

Therefore, I invite you to prepare and submit a revised version of your manuscript. Please address all the reviewers' comments in detail, and include a point-by-point response to their critiques in your cover letter.

I look forward to receiving your revised manuscript.

Please submit your revised manuscript by Nov 08 2025 11:59PM. If you will need more time than this to complete your revisions, please reply to this message or contact the journal office at plosone@plos.org. When you're ready to submit your revision, log on to https://www.editorialmanager.com/pone/ and select the 'Submissions Needing Revision' folder to locate your manuscript file.

Please include the following items when submitting your revised manuscript:

- A rebuttal letter that responds to each point raised by the academic editor and reviewer(s). You should upload this letter as a separate file labeled 'Response to Reviewers'.
- A marked-up copy of your manuscript that highlights changes made to the original version. You should upload this as a separate file labeled 'Revised Manuscript with Track Changes'.
- An unmarked version of your revised paper without tracked changes. You should upload this as a separate file labeled 'Manuscript'.

If you would like to make changes to your financial disclosure, please include your updated statement in your cover letter. Guidelines for resubmitting your figure files are available below the reviewer comments at the end of this letter.

If applicable, we recommend that you deposit your laboratory protocols in protocols.io to enhance the reproducibility of your results. Protocols.io assigns your protocol its own identifier (DOI) so that it can be cited independently in the future. For instructions see: https://journals.plos.org/plosone/s/submission-guidelines#loc-laboratory-protocols. Additionally, PLOS ONE offers an option for publishing peer-reviewed Lab Protocol articles, which describe protocols hosted on protocols.io. Read more information on sharing protocols at https://plos.org/protocols?utm_medium=editorial-email&utm_source=authorletters&utm_campaign=protocols.

We look forward to receiving your revised manuscript.

Kind regards,

Jie Zeng, Ph.D.

Academic Editor

PLOS ONE

Journal Requirements:

When submitting your revision, we need you to address these additional requirements.

1. Please ensure that your manuscript meets PLOS ONE's style requirements, including those for file naming. The PLOS ONE style templates can be found at

https://journals.plos.org/plosone/s/file?id=wjVg/PLOSOne_formatting_sample_main_body.pdf and

https://journals.plos.org/plosone/s/file?id=ba62/PLOSOne_formatting_sample_title_authors_affiliations.pdf

2. Thank you for stating the following financial disclosure:

“Anhui Provincial Department of Education Cultivation Program for Excellent and Top-notch Talents in Colleges and Universities (Key Project of Support Program for Excellent Young Talents in Colleges and Universities) (Number:gxyqZD2022087)；Anhui Provincial Department of Education 2023 Annual College Ancient Book Arrangement Research Project (Number:2023AHGJ04)；Anhui College of the Arts “Classroom Excellence” Project: Ancient Genealogy Poetry Creation and Performance Planning (Number:2025xjzykt11).”

Please state what role the funders took in the study. If the funders had no role, please state: "The funders had no role in study design, data collection and analysis, decision to publish, or preparation of the manuscript."

If this statement is not correct you must amend it as needed.

Please include this amended Role of Funder statement in your cover letter; we will change the online submission form on your behalf.

3. We note that your Data Availability Statement is currently as follows: All relevant data are within the manuscript and its Supporting Information files.

Please confirm at this time whether or not your submission contains all raw data required to replicate the results of your study. Authors must share the “minimal data set” for their submission. PLOS defines the minimal data set to consist of the data required to replicate all study findings reported in the article, as well as related metadata and methods (https://journals.plos.org/plosone/s/data-availability#loc-minimal-data-set-definition).

For example, authors should submit the following data:

- The values behind the means, standard deviations and other measures reported;

- The values used to build graphs;

- The points extracted from images for analysis.

Authors do not need to submit their entire data set if only a portion of the data was used in the reported study.

If your submission does not contain these data, please either upload them as Supporting Information files or deposit them to a stable, public repository and provide us with the relevant URLs, DOIs, or accession numbers. For a list of recommended repositories, please see https://journals.plos.org/plosone/s/recommended-repositories.

If there are ethical or legal restrictions on sharing a de-identified data set, please explain them in detail (e.g., data contain potentially sensitive information, data are owned by a third-party organization, etc.) and who has imposed them (e.g., an ethics committee). Please also provide contact information for a data access committee, ethics committee, or other institutional body to which data requests may be sent. If data are owned by a third party, please indicate how others may request data access.

4. PLOS requires an ORCID iD for the corresponding author in Editorial Manager on papers submitted after December 6th, 2016. Please ensure that you have an ORCID iD and that it is validated in Editorial Manager. To do this, go to ‘Update my Information’ (in the upper left-hand corner of the main menu), and click on the Fetch/Validate link next to the ORCID field. This will take you to the ORCID site and allow you to create a new iD or authenticate a pre-existing iD in Editorial Manager.

5. Please include your full ethics statement in the ‘Methods’ section of your manuscript file. In your statement, please include the full name of the IRB or ethics committee who approved or waived your study, as well as whether or not you obtained informed written or verbal consent. If consent was waived for your study, please include this information in your statement as well.

6. We note that Figure 2 includes an image of a participant.

As per the PLOS ONE policy (http://journals.plos.org/plosone/s/submission-guidelines#loc-human-subjects-research) on papers that include identifying, or potentially identifying, information, the individual(s) or parent(s)/guardian(s) must be informed of the terms of the PLOS open-access (CC-BY) license and provide specific permission for publication of these details under the terms of this license. Please download the Consent Form for Publication in a PLOS Journal (http://journals.plos.org/plosone/s/file?id=8ce6/plos-consent-form-english.pdf). The signed consent form should not be submitted with the manuscript, but should be securely filed in the individual's case notes. Please amend the methods section and ethics statement of the manuscript to explicitly state that the patient/participant has provided consent for publication: “The individual in this manuscript has given written informed consent (as outlined in PLOS consent form) to publish these case details”.

Please respond by return e-mail with an amended manuscript. We can upload this to your submission on your behalf.

If you are unable to obtain consent from the subject of the photograph, please either instruct us to remove the figure or supply a replacement figure by return e-mail for which you hold the relevant copyright permissions and subject consents. In some cases, you may need to specify in the text that the image used in the figure is not the original image used in the study, but a similar image used for illustrative purposes only. We can make any changes on your behalf.

7. Please include captions for your Supporting Information files at the end of your manuscript, and update any in-text citations to match accordingly. Please see our Supporting Information guidelines for more information: http://journals.plos.org/plosone/s/supporting-information.

8. If the reviewer comments include a recommendation to cite specific previously published works, please review and evaluate these publications to determine whether they are relevant and should be cited. There is no requirement to cite these works unless the editor has indicated otherwise.

[Note: HTML markup is below. Please do not edit.]

Reviewers' comments:

Reviewer's Responses to Questions

**Comments to the Author**

1. Is the manuscript technically sound, and do the data support the conclusions?

The manuscript must describe a technically sound piece of scientific research with data that supports the conclusions. Experiments must have been conducted rigorously, with appropriate controls, replication, and sample sizes. The conclusions must be drawn appropriately based on the data presented.

Reviewer #1: Yes

Reviewer #2: Partly

**********

2. Has the statistical analysis been performed appropriately and rigorously?

Reviewer #1: No

Reviewer #2: No

**********

3. Have the authors made all data underlying the findings in their manuscript fully available?

The PLOS Data policy requires authors to make all data underlying the findings described in their manuscript fully available without restriction, with rare exception (please refer to the Data Availability Statement in the manuscript PDF file). The data should be provided as part of the manuscript or its supporting information, or deposited to a public repository. For example, in addition to summary statistics, the data points behind means, medians and variance measures should be available. If there are restrictions on publicly sharing data—e.g. participant privacy or use of data from a third party—those must be specified.

Reviewer #1: Yes

Reviewer #2: No

**********

4. Is the manuscript presented in an intelligible fashion and written in standard English?

PLOS ONE does not copyedit accepted manuscripts, so the language in submitted articles must be clear, correct, and unambiguous. Any typographical or grammatical errors should be corrected at revision, so please note any specific errors here.

Reviewer #1: No

Reviewer #2: Yes

**********

5. Review Comments to the Author

Please use the space provided to explain your answers to the questions above. You may also include additional comments for the author, including concerns about dual publication, research ethics, or publication ethics. (Please upload your review as an attachment if it exceeds 20,000 characters)

Reviewer #1: “The manuscript employs the ‘leisure constraints/constraints–negotiation’ framework to examine constraints on the experience of ancient-score poetry, combining audience surveys from three ‘Wei’s Scores’ concerts and analyzing the data with PLS-SEM. The topic is innovative and relevant to cultural heritage; the materials are novel; and preliminary results suggest pathways through which intrapersonal and structural constraints affect preference and participation, offering some academic and practical value. However, there is considerable room for improvement in research design transparency, scale development and validation, statistical reporting standards, language and scholarly expression, and compliance with ethics and data sharing. A major revision is recommended before re-review.

1. Insufficient standardization and testability of research questions and hypotheses

The research hypotheses are listed at the end of Section 2 but are inconsistent with the model annotations, and it is not stated whether ‘negotiation’ was measured, indicating a theory–measurement inconsistency (lines 117–136, 137–138). Recommendation: Consolidate testable hypotheses at the end of the Introduction or the start of Methods, ensure one-to-one correspondence with the paths in Figure 1, and standardize terminology boundaries (preference/participation/satisfaction and the three types of constraints).

2. Scale development and psychometric evidence need to be completed and standardized The origins of the scales and the item development process are not described. For example, there is no information on the item pool, proportions of adapted vs. self-developed items, expert review, pilot testing, or item screening criteria (lines 189–197). The measurement model reporting is incomplete. The paper provides only some loadings, α, CR, AVE, and VIF. Please add the full item lists for each latent construct, means/variances of standardized loadings, 95% CIs for α/CR/AVE, HTMT and the cross-loading matrix; explain any item deletions and the reasons (lines 226–239, 240–247, and the pages containing Tables 2 and 3, lines 229–246). Construct operationalization shows domain drift. Participation includes items like “number of classical concerts,” which are not fully domain-consistent with “participation in ancient-score poetry”; see Table 2 items “Your average number of concerts per year” and “The average number of times you listen to classical music concerts per year.” Scale language quality issues. There is substantial Chinglish and unnatural phrasing that affects comprehension and the interval-scale assumption, appearing throughout the manuscript, notably in the abstract and the items in Table 2 (lines 7–29, 229–246).

3. Sampling/procedure transparency and representativeness Sampling and procedures are unclear. The sources of audiences for the three concerts, inclusion/exclusion criteria, recruitment channels, online/offline questionnaire ratio, and quality control (de-duplication, response time, attention checks) are not specified (lines 140–161, 164–187, 194–201). Remove Figure 2; it does not support the study. Sample representativeness issues. Military personnel account for 44.4%, indicating significant bias; the 300 general-public respondents recruited via the official WeChat account constitute a self-selected sample. The boundaries of external validity should be honestly stated and discussed (lines 164–175, 209–223, and p. 24, lines 173–187).

4. PLS-SEM analysis and reporting standards The rationale for choosing PLS is insufficient. Justify based on sample size, model complexity, and a prediction-oriented goal, and specify software version, algorithm, number of bootstrap resamples, and type of confidence interval (lines 226–236). Please add that SmartPLS 4 was used, and provide the missing details. Structural model quality indicators are incomplete. Q², f², PLSpredict, and VIFs for endogenous independent variables are missing; interpreting R² > 0.02 as “high” is inappropriate—especially for Satisfaction where R² = 0.038, which calls for discussion of measurement or theoretical reasons (lines 267–273, Table 4). Path coefficients are inconsistent. In the figure, Structural → Participate is −0.086, while Table 5 reports −0.28. Unify these based on bootstrap statistics; Figure 3 still contains residual phrases like “Assuming it’s true/Assumptions are not valid” (lines 237–239; Table 5; lines 274–276; pp. 20–21).

5. Causal and generalization claims in Results/Discussion Causal caution is insufficient. Given a convenience sample and no control design, the paths represent covariation only; avoid causal and policy-oriented extrapolations. The abstract, discussion, and conclusion contain multiple overstated claims (lines 7–27, 275–291, 443–472, 480–497). Mechanisms and mediation. The Participation → Satisfaction path is non-significant, while Preference → Participation is significant. Test the indirect effect or moderation of Preference, and acknowledge that the unmeasured ‘negotiation’ dimension represents a theory–measurement gap (lines 254–266, 274–276).

6. Writing and scholarly expression There is Chinglish and mixed terminology in the title, abstract, and throughout (e.g., “Leisure Restricted Class Modeling,” “ancient genealogy/ancient spectrum”); professional English editing and terminological unification are recommended (short title and keywords on p. 1; lines 7–29; items in lines 189–201). Avoid promotional statements beyond the study’s scope (e.g., “music therapy”); trim grand narratives and slogan-like passages, and focus on evidence-supported claims (lines 335–353, 488–497). The references contain duplicates, author–year inconsistencies, and incomplete translation/journal details. Standardize formatting per the journal’s English style and deduplicate (lines 548–551, 570–573, 600–607, 612–616).”

Reviewer #2: The manuscript explores the integration of ancient Chinese poetry with musical performance and immersive art activities—an innovative and culturally meaningful endeavor. Collaborations with museums, along with the use of guided listening sessions, workshops, and modern technology, enhance the value of the study. A reasonable number of valid survey responses were collected, and the application of Smart- PLS 4 to assess reliability and validity represents a solid analytical approach.

However, several methodological limitations should be noted. The sampling strategy, which relied exclusively on concert attendees, may compromise representativeness and introduce self-selection bias. Although factor loadings, Cronbach’s alpha, composite reliability (CR), and average variance extracted (AVE) were reported, the threshold of α > 0.6 is considered lenient, and evidence for discriminant validity remains insufficient. More rigorous statistical reporting—including hypothesis testing and effect sizes—would strengthen the analysis. The absence of a control group also limits the ability to make causal inferences. Furthermore, in accordance with PLOS data policy, it is unclear whether the complete raw data have been made publicly available; this should be explicitly addressed.

The manuscript is clearly written and generally in acceptable English, though some sections could benefit from improved conciseness and clarity. Overall, the study shows promise, but revisions focusing on methodological rigor, statistical depth, and data transparency would enhance its scholarly contribution.

**********

6. PLOS authors have the option to publish the peer review history of their article (what does this mean?). If published, this will include your full peer review and any attached files.

If you choose “no”, your identity will remain anonymous but your review may still be made public.

**Do you want your identity to be public for this peer review?** For information about this choice, including consent withdrawal, please see our Privacy Policy.

Reviewer #1: No

Reviewer #2: No

**********

[NOTE: If reviewer comments were submitted as an attachment file, they will be attached to this email and accessible via the submission site. Please log into your account, locate the manuscript record, and check for the action link "View Attachments". If this link does not appear, there are no attachment files.]

While revising your submission, please upload your figure files to the Preflight Analysis and Conversion Engine (PACE) digital diagnostic tool, https://pacev2.apexcovantage.com/. PACE helps ensure that figures meet PLOS requirements. To use PACE, you must first register as a user. Registration is free. Then, login and navigate to the UPLOAD tab, where you will find detailed instructions on how to use the tool. If you encounter any issues or have any questions when using PACE, please email PLOS at figures@plos.org. Please note that Supporting Information files do not need this step.

Attachment Submitted filename: PONE-D-25-36046.pdf

# Author comment: Author response to Decision Letter 1

**DOI:** 10.1371/journal.pone.0346421.r002

21 Jan 2026

Dear editor:

We sincerely appreciate the valuable comments provided by the reviewers. All modifications made in the manuscript are highlighted in blue.

Journal Requirements:

1.Editorial comments：Please ensure that your manuscript complies with the style requirements of PLOS ONE, including the requirements for file naming.

Our response：We are grateful for the valuable comments from the reviewers. We have revised the article format again in accordance with the requirements of the journal.

2.Editorial comments：Please describe the role of the funder in the research. If the funder had no role, state: “The funder had no role in the research design, data collection and analysis, publication decisions, or manuscript preparation.”

Our response：Thank you for the valuable comments from the reviewers. We have re-stated the role of the funder. Anhui Provincial Department of Education Cultivation Program for Excellent and Top-notch Talents in Colleges and Universities (Key Project of Support Program for Excellent Young Talents in Colleges and Universities) (Number:gxyqZD2022087)；Anhui Provincial Department of Education 2023 Annual College Ancient Book Arrangement Research Project (Number:2023AHGJ04)；Anhui College of the Arts “Classroom Excellence” Project: Ancient Genealogy Poetry Creation and Performance Planning (Number:2025xjzykt11).

(ZS served as the principal investigator for project gxyqZD2022087 and the second contributor for project 2023AHGJ04, while PJ was the principal investigator for project 2025xjzykt11. Both made significant contributions to research design, data collection, and analysis.)

3. Editorial comments ：We note that your data availability statement currently reads: All relevant data are included in the manuscript and its supporting information files.

Our response：Please confirm at this time whether your submission contains all the raw data necessary to replicate your research findings. Authors must share the “minimal dataset” for their submission. PLOS defines the minimal dataset as including all data required to replicate every research finding reported in the article, along with associated metadata and methods.

4. Editorial comments：PLOS requires that manuscripts submitted after December 6, 2016, provide the corresponding author's ORCID iD in Editorial Manager.

Our response：Thank you for the valuable comments from the reviewers. We have updated the ORCID ID of the corresponding author again.

5.Editorial comments：Please include your complete ethics statement in the “Methods” section of your manuscript. In your statement, include the full name of the IRB or ethics committee that approved or waived your research, and whether you obtained informed written or verbal consent. If consent was waived for your research, please also include this information in your statement.

Our response：We appreciate the reviewers' valuable feedback and have added the ethics committee statement and informed consent form to the Methods section.

6.Editorial comments：We note that Figure 2 includes images of participants…….

Our response：We appreciate the reviewers' comments. All images used in this study have been obtained with participants' consent and informed consent forms have been signed. Furthermore, our questionnaire does not involve any privacy concerns of the respondents. All procedures in this study comply with Anhui University's research guidelines and the 1964 Declaration of Helsinki and its subsequent amendments or equivalent ethical standards. Survey responses will be collected anonymously via Questionnaire Star and paper questionnaires. Informed consent was obtained from all participants and their guardians upon distribution of the questionnaires.

7.Editorial comments：Please add the title of the supporting information file at the end of your manuscript and update any text citations accordingly. For more information, please refer to our Supporting Information Guidelines:

Our response：We have resubmitted the supporting documentation in response to the reviewers' comments.

8.Editorial comments： If a reviewer's comments include suggestions to cite specific previously published works, please review and evaluate these publications to determine whether they are relevant and should be referenced. Unless otherwise specified by the editor, there is no requirement to cite these works.

Our response：Alright, thank you for your valuable feedback.

Reviewer 1 Comments:

This manuscript employs a ‘leisure constraint/constraint-negotiation’ framework to examine constraints on the experience of ancient musical notation poetry. It integrates audience surveys from three ‘Wei's Musical Notation’ concerts and utilizes PLS-SEM to analyze the data. The topic is innovative and relevant to cultural heritage; the materials are novel. Preliminary findings suggest pathways through which individual internal and structural constraints influence preferences and engagement, offering academic and practical value. However, significant improvements are needed in research design transparency, scale development and validation, statistical reporting standards, language and scholarly expression, and adherence to ethics and data sharing. Substantive revisions are recommended prior to reconsideration.

1. Insufficient Standardization and Testability of Research Questions and Hypotheses Research hypotheses are listed at the end of Section 2 but are inconsistent with model annotations. Furthermore, it remains unclear whether “negotiation” is measured, indicating a disconnect between theory and measurement (Lines 117-136, Lines 137-138). Recommendation: Consolidate testable hypotheses at the end of the Introduction or beginning of the Methods section, ensuring one-to-one correspondence with paths in Figure 1 and standardizing boundary terms (preference/engagement/satisfaction and three types of constraints).

Our Response and Revisions:

We appreciate the reviewer's comments and have carefully incorporated the suggested revisions into the manuscript. Specific changes are outlined below:

Added the corresponding hypothesis for Figure 1 at the end of the Introduction: By exploring constraints on the “three-dimensional creation” experience and integrating the “leisure constraints” theory (Gilbert and Hudson, 2000; Hung and Petrick, 2010), we propose the following hypothesis: “Personal constraints → Preference → Negative, interpersonal relationships → Participation → Negative, structural constraints → Participation → Negative, interpersonal relationships → Satisfaction → Negative, structural constraints → Satisfaction → Negative.” We also investigate the relationship between “Preference → Participation → Positive, Participation → Satisfaction → Positive.”（Lines 43-56）.

Theoretical Basis for Scale Revision: This study designed and revised the scale for measuring experiential constraints in classical poetry appreciation based on Churchill's scale design specifications, adapting it to experiential contexts (Gilbert and Hudson, 2000; Hung and Petrick, 2010)（Lines 103-111）.

Reviewer1 Comments:

2. Scale development and psychometric evidence require completion and standardization. The scale's origin and item development process are not described. For example, there is no information regarding the item pool, the ratio of adapted items to self-developed items, expert review, pilot testing, or item screening criteria (lines 189-197). The measurement model report is incomplete. The paper only provides some loadings, namely α, CR, AVE, and VIF. Please add: - A complete item list for each latent construct - Mean/variance of standardized loadings - 95% CIs for α/CR/AVE - HTMT and cross-loading matrices - Explanations for any item deletions and their reasons (Lines 226-239, Lines 240-247, and pages containing Tables 2 and 3, Lines 229-246). The construct operationalization exhibits domain drift. Items such as “number of classical concerts attended” are not fully consistent with “participation in ancient musical poetry”; see Table 2 items “Your average number of concerts per year” and “Your average number of classical music concerts attended per year.” Expanded language quality issues. Significant amounts of Chinglish and unnatural phrasing affecting comprehension and pitch scale assumptions appear throughout the manuscript, particularly in items within the abstract and Table 2 (lines 7-29, 229-246).

Reviewer Comments: Scale development and psychometric evidence require completion and standardization. The scale's origin and item development process are not described. For example, there is no information regarding the item pool, the ratio of adapted items to self-developed items, expert review, pilot testing, or item selection criteria.

Our Response and Revisions:

We sincerely appreciate the reviewer's valuable comments. We have carefully incorporated your suggestions into the manuscript with the following specific revisions:

The scale design for measuring classical poetry experience constraints, based on Churchill's (1979) measurement scale design specifications, and its revision within the experiential context (Gilbert and Hudson, 2000; Hung and Petrick, 2010). Considering the unique characteristics of classical poetry appreciation, we developed a 22-item scale. Following expert panel discussions and preliminary online survey results, two items with low relevance and selection frequency were removed, resulting in a final 20-item scale for measuring classical poetry appreciation. This scale suggests relationships exist between the constraint dimension and preferences, engagement, and satisfaction with classical poetry.（Lines 103-111）

Reviewer comments: This table provides only a few loadings, namely α, CR, AVE, and VIF. Please add a complete item list for each latent construct, the mean/variance of standardized loadings, 95% CIs for α/CR/AVE, HTMT, and cross-loading matrices; explain any item deletions and their reasons.

Our Response and Revisions:

We sincerely apologize for the previous incomplete report. We have systematically supplemented all requested metrics to ensure comprehensiveness in the measurement model evaluation.

1. Complete Item Lists: We have provided complete item lists for all constructs in Tables 4 and 5.

2. Supplementary Statistical Metrics:

We report the mean and variance of standardized loadings for each construct in the revised Table 2. We calculated 95% bias-corrected confidence intervals for α, CR, and AVE using Bootstrap (5000 samples) and reported them in the measurement model results section or table footnotes. We supplemented the Heterogeneity-to-Homogeneity Ratio (HTMT) matrix as key evidence for discriminant validity (see Table 4), with all values below the conservative threshold of 0.85. We supplemented cross-loadings matrices and means/standard deviations (see Table 5), showing all items loaded higher on their target construct than on other constructs.

Henseler, J., Ringle, C. M., & Sarstedt, M. (2015). A new criterion for assessing discriminant validity in variance-based structural equation modeling. Journal of the Academy of Marketing Science, 43 (1), 115–135.https://doi.org/10.1007/s11747-014-0403-8

Item Deletion Explanation: As described in the newly added scale development process above, no items were deleted during the item analysis following the pilot test because all items met the predetermined retention criteria. This has been explicitly stated in the text.

Table 4. HTMT Evaluation

Project Personal constraints preference Interpersonal constraints Structural constraints Participate Satisfaction

Personal constraints 1.000 0.249 0.520 0.456 0.194 0.254

preference 0.249 1.000 0.271 0.290 0.386 0.348

Interpersonal constraints 0.520 0.271 1.000 0.750 0.357 0.173

Structural constraints 0.456 0.290 0.750 1.000 0.412 0.178

Participate 0.194 0.386 0.357 0.412 1.000 0.132

Satisfaction 0.254 0.348 0.173 0.178 0.132 1.000

Table 5. Cross-Load Matrix

Project Personal constraints Interpersonal constraints preference Participate Satisfaction Structural constraints average value Standard deviation

interest 0.805 0.371 0.231 0.108 -0.24 0.338 1.976 0.943

poems 0.88 0.453 0.209 0.185 0.187 0.393 2.139 0.963

melodic 0.844 0.488 0.184 0.206 0.212 0.428 2.158 0.968

thing 0.399 0.69 -0.31 0.367 -0.14 0.785 3.08 1.125

work 0.372 0.53 0.213 0.231 0.193 0.782 2.764 1.188

time 0.383 0.557 0.241 0.300 0.148 0.809 2.783 1.154

information 0.356 0.679 0.273 0.430 -0.11 0.875 3.049 1.17

spot 0.294 0.498 0.081 0.274 0.123 0.7 2.9 1.093

companions 0.502 0.832 0.198 0.290 0.193 0.549 2.384 1.052

tuition 0.322 0.843 0.229 0.339 0.086 0.696 3.068 1.167

accompany 0.432 0.749 0.239 0.233 0.136 0.595 2.596 1.189

daily focus 0.237 0.265 0.921 0.385 0.301 0.287 3.869 1.378

travel matters 0.211 0.221 0.881 0.305 0.328 0.235 4.212 1.378

realize 0.138 0.247 0.246 0.715 0.123 0.308 2.676 0.891

concerts 0.186 0.217 0.328 0.757 0.143 -0.25 2.759 1.041

ancient records 0.124 0.348 0.31 0.814 0.042 0.386 1.903 0.912

overall rating 0.225 0.131 0.194 -0.03 0.773 0.086 4.328 0.775

watch again 0.209 0.145 0.323 0.14 0.914 0.137 4.304 0.827

friend 0.202 0.157 0.341 0.106 0.919 0.167 4.294 0.788

wish 0.266 0.164 0.322 0.165 0.911 0.193 4.35 0.754

Reviewer Comments: Sampling/Procedure Transparency and Representativeness Sampling and procedures were not clearly defined. The sources of audiences for the three concerts, inclusion/exclusion criteria, recruitment channels, online/offline questionnaire ratios, and quality control measures (duplicate data removal, response time, attention checks) were not specified (Lines 140-161, Lines 164-187, Lines 194-201). Remove Figure 2; it does not support this study. Sample representativeness issues. Military personnel constituted 44.4%, indicating significant bias; the 300 public respondents recruited via an official WeChat public account formed a self-selected sample. The limitations to external validity should be honestly stated and discussed (Lines 164–175, 209–223, and 164–175, 223, 164–175, 223, 164–175, 209–223, 164–175, 223, 164–24, 173–187).

Our Response and Revisions:

We sincerely appreciate your meticulous review of our manuscript and for highlighting this critical data issue. You are absolutely correct—the initial report indicating that military personnel constituted 44.4% of the sample indeed revealed a significant bias in the data.（Table 1 Descriptive Statistics） We sincerely apologize for this data error and have conducted a thorough review and correction. Upon investigation, the error stemmed from a misstep in the translation sequence between Chinese and English. During the data cleaning phase, due to classification issues between paper and electronic questionnaires, students were mistakenly categorized as “military personnel” during translation. We have re-examined the raw data and implemented a rigorous data validation process.（Lines 205-216）

Variable Category Frequency proportion

Your occupation civil servant 17 4.1

Enterprise and institutional managers 46 11.1

Professional/Technical and Educational Personnel 99 24

Service Sales and Trade Personnel 5 1.2

Worker 2 0.4

peasant 0 0

Military personnel 2 0.4

student 176 42.8

Retirees 15 3.6

Other occupations 49 11.9

Furthermore, we have detailed the recruitment process and quality control guidelines in the main text (line 191-203):

Data for this study was collected during three ancient poetry concerts held between August and December 2024. To ensure sample openness and diversity, audiences for the latter two concerts were recruited via the official WeChat public account using a first-come-first-served random reservation model. Each concert automatically closed registration once 300 attendees were reached, eliminating any manual selection. To accommodate audience habits across different age groups—particularly avoiding a digital divide for students and seniors—data collection employed a combination of paper and electronic questionnaires. Across the three surveys, 322 paper questionnaires were distributed with 281 returned, while 226 electronic questionnaires were simultaneously collected. After rigorous quality control of all returned data—including deduplication based on demographic information, screening response times, and

Attachment Submitted filename: Response to Reviewers.docx

# Peer review: Decision Letter 1

**DOI:** 10.1371/journal.pone.0346421.r003

**Authors:** Jie Zeng

19 Mar 2026

A Study of the Limitations of Musical Experience in Ancient Chinese Poetry—The Case of the Creation Concert of Wei's Music Score

PONE-D-25-36046R1

Dear Dr. Panjie Xie,

We’re pleased to inform you that your manuscript has been judged scientifically suitable for publication and will be formally accepted for publication once it meets all outstanding technical requirements.

Within one week, you’ll receive an e-mail detailing the required amendments. When these have been addressed, you’ll receive a formal acceptance letter and your manuscript will be scheduled for publication.

An invoice will be generated when your article is formally accepted. Please note, if your institution has a publishing partnership with PLOS and your article meets the relevant criteria, all or part of your publication costs will be covered. Please make sure your user information is up-to-date by logging into Editorial Manager at Editorial Manager® and clicking the ‘Update My Information' link at the top of the page. For questions related to billing, please contact billing support.

If your institution or institutions have a press office, please notify them about your upcoming paper to help maximize its impact. If they’ll be preparing press materials, please inform our press team as soon as possible -- no later than 48 hours after receiving the formal acceptance. Your manuscript will remain under strict press embargo until 2 pm Eastern Time on the date of publication. For more information, please contact onepress@plos.org.

Kind regards,

Jie Zeng, Ph.D.

Academic Editor

PLOS One

Additional Editor Comments (optional):

The third reviewer has recommended acceptance; however, he or she noted that some minor revisions are still required. Please carefully revise your manuscript accordingly before submitting the final version.

Reviewers' comments:

Reviewer's Responses to Questions

**Comments to the Author**

1. If the authors have adequately addressed your comments raised in a previous round of review and you feel that this manuscript is now acceptable for publication, you may indicate that here to bypass the “Comments to the Author” section, enter your conflict of interest statement in the “Confidential to Editor” section, and submit your "Accept" recommendation.

Reviewer #1: (No Response)

Reviewer #2: All comments have been addressed

Reviewer #3: All comments have been addressed

**********

2. Is the manuscript technically sound, and do the data support the conclusions?

The manuscript must describe a technically sound piece of scientific research with data that supports the conclusions. Experiments must have been conducted rigorously, with appropriate controls, replication, and sample sizes. The conclusions must be drawn appropriately based on the data presented.

Reviewer #1: No

Reviewer #2: Yes

Reviewer #3: Yes

**********

3. Has the statistical analysis been performed appropriately and rigorously?

Reviewer #1: No

Reviewer #2: Yes

Reviewer #3: Yes

**********

4. Have the authors made all data underlying the findings in their manuscript fully available?

The PLOS Data policy requires authors to make all data underlying the findings described in their manuscript fully available without restriction, with rare exception (please refer to the Data Availability Statement in the manuscript PDF file). The data should be provided as part of the manuscript or its supporting information, or deposited to a public repository. For example, in addition to summary statistics, the data points behind means, medians and variance measures should be available. If there are restrictions on publicly sharing data—e.g. participant privacy or use of data from a third party—those must be specified.

Reviewer #1: (No Response)

Reviewer #2: Yes

Reviewer #3: Yes

**********

5. Is the manuscript presented in an intelligible fashion and written in standard English?

PLOS ONE does not copyedit accepted manuscripts, so the language in submitted articles must be clear, correct, and unambiguous. Any typographical or grammatical errors should be corrected at revision, so please note any specific errors here.

Reviewer #1: (No Response)

Reviewer #2: Yes

Reviewer #3: Yes

**********

6. Review Comments to the Author

Please use the space provided to explain your answers to the questions above. You may also include additional comments for the author, including concerns about dual publication, research ethics, or publication ethics. (Please upload your review as an attachment if it exceeds 20,000 characters)

Reviewer #1: Thank you for the revised submission and the detailed point-by-point response. While I appreciate the effort to address reviewer and editorial concerns, I do not think the manuscript reaches the methodological and reporting standards required for publication in PLOS ONE. My recommendation is reject, primarily due to persistent internal inconsistencies, unresolved theory–measurement misalignment, and incomplete compliance with key journal requirements.

1) Theory–measurement misalignment remains unresolved

The manuscript is framed within a “leisure constraints/constraint-negotiation” tradition, yet it remains unclear whether negotiation is actually measured or modeled. This represents a fundamental disconnect between the stated theoretical framework and the implemented measurement/structural model. In the response you acknowledge this gap but do not correct it by either (a) measuring negotiation and testing its role (including indirect effects/mediation as suggested), or (b) revising the theoretical framing and claims so that negotiation is not presented as part of the explanatory mechanism. This problem affects interpretability of the entire model.

2) Scale development and construct validity are still not sufficiently transparent or convincing

You state that the constraints scale was developed using Churchill (1979) procedures and reduced from 22 to 20 items after expert discussion and a preliminary survey. However, the manuscript still lacks essential details needed for reproducibility and psychometric evaluation (e.g., item pool generation process, proportion of adapted vs. newly developed items, expert panel composition/criteria, pilot sample size, explicit decision rules for deletion beyond “low relevance and selection frequency”).

More importantly, the domain drift problem for “Participation” remains: items such as “your average number of concerts per year” and “the average number of times you listen to classical music concerts per year” do not clearly operationalize participation in ancient musical-notation poetry experiences and therefore threaten content validity. This issue was explicitly raised and has not been substantively resolved.

3) Statistical reporting problems raise concerns about result credibility

Although the response claims that reporting has been standardized and that additional indices (e.g., Q², f², VIF) were added, the revised materials provided still show major presentation problems that prevent verification. For example, the discriminant validity table appears corrupted/misaligned and is not interpretable as presented. When core validity evidence cannot be clearly checked, the measurement model cannot be evaluated to PLOS ONE standards.

In addition, you describe bootstrap settings in the response (e.g., 10,000 subsamples, BCa CIs), but the manuscript needs to be fully consistent and transparent about the exact estimation settings used for all reported results.

4) Writing and scholarly tone remain problematic in key places

Despite claims of professional editing, the abstract and parts of the manuscript still contain highly promotional, overgeneralized claims and non-standard phrasing that undermine scholarly clarity (e.g., assertions about cultural self-confidence and broad societal impacts not directly supported by the model and sampling approach). The paper would require substantial rewriting for precision, cautious inference, and terminology standardization.

Reviewer #2: The authors have demonstrated exceptional academic rigor in addressing the feedback from the previous review cycle. The manuscript has significantly evolved from its original version into a technically sound and well-structured study that bridges "Leisure Constraints Theory" with the preservation of cultural heritage. Key Technical Improvements: Regarding statistical robustness, the authors have provided comprehensive evidence for the PLS-SEM analysis, including necessary metrics such as HTMT, VIF, $f^2$, and $Q^2$. The use of 5,000 Bootstrap runs with BCa confidence intervals ensures the stability and reliability of the path coefficients. In terms of data integrity and transparency, the rectification of demographic data (correcting the student vs. military classification error) and the detailed description of the sampling procedure, specifically the random reservation model, have resolved previous concerns regarding data bias and representativeness. Furthermore, the conceptual alignment has been strengthened as the research hypotheses are now clearly defined and show a one-to-one correspondence with the structural model, effectively addressing the "negotiation" measurement gap identified previously. Scholarly Presentation: The manuscript has undergone professional editing, resulting in clear and unambiguous academic English. The removal of nearly 1,000 words of non-academic narrative has significantly enhanced the professional tone and flow of the paper. Regarding discussion depth, the authors have appropriately re-evaluated the $R^2$ values and adopted a more cautious approach toward causal inferences, acknowledging the inherent limitations of cross-sectional field studies.

Reviewer #3: Overall Evaluation

This study applies leisure constraints theory to ancient poetry experience, and the topic is interesting. The research methods are appropriate, the data analysis is generally sound, and the overall structure is clear. However, the manuscript still needs improvement in methodological reporting and result interpretation. Below are specific suggestions for the final polishing stage.

Suggestions

1. Sample size justification

The authors did not explain how the sample size was determined. PLS-SEM typically follows the 10-times rule, which means the sample should exceed 10 times the largest number of paths pointing to a single construct. Please clarify whether the sample of 411 meets this requirement.

2. Causal language

The manuscript uses the word “influence” in several sections. Because this study relies on cross-sectional data, it can only demonstrate correlations rather than direct causality. Please replace “influence” with terms such as “predict” or “correlate with” in some expressions.

3. Model explanatory power

The R² values for preference (0.062) and satisfaction (0.038) are low. The authors’ explanation is reasonable, but the discussion could be expanded to acknowledge potential omitted variables, such as cultural capital or prior poetry exposure.

4. Sentence length

Some sentences are too long. Breaking them into shorter sentences would improve readability.

Summary

The study is methodologically sound and the conclusions are credible. With improvements in reporting, interpretation, and language, the manuscript will be stronger. Please make these revisions during the final polishing stage.

**********

7. PLOS authors have the option to publish the peer review history of their article (what does this mean?). If published, this will include your full peer review and any attached files.

If you choose “no”, your identity will remain anonymous but your review may still be made public.

**Do you want your identity to be public for this peer review?** For information about this choice, including consent withdrawal, please see our Privacy Policy.

Reviewer #1: No

Reviewer #2: No

Reviewer #3: No

**********

Attachment Submitted filename: Review Comments Manuscript ID [PONE-D-25-36046R1].docx

# Editor report: Acceptance letter

**DOI:** 10.1371/journal.pone.0346421.r004

**Authors:** Jie Zeng

PONE-D-25-36046R1

PLOS One

Dear Dr. Xie,

I'm pleased to inform you that your manuscript has been deemed suitable for publication in PLOS One. Congratulations! Your manuscript is now being handed over to our production team.

At this stage, our production department will prepare your paper for publication. This includes ensuring the following:

* All references, tables, and figures are properly cited

* All relevant supporting information is included in the manuscript submission,

* There are no issues that prevent the paper from being properly typeset

You will receive further instructions from the production team, including instructions on how to review your proof when it is ready. Please keep in mind that we are working through a large volume of accepted articles, so please give us a few days to review your paper and let you know the next and final steps.

Lastly, if your institution or institutions have a press office, please let them know about your upcoming paper now to help maximize its impact. If they'll be preparing press materials, please inform our press team within the next 48 hours. Your manuscript will remain under strict press embargo until 2 pm Eastern Time on the date of publication. For more information, please contact onepress@plos.org.

You will receive an invoice from PLOS for your publication fee after your manuscript has reached the completed accept phase. If you receive an email requesting payment before acceptance or for any other service, this may be a phishing scheme. Learn how to identify phishing emails and protect your accounts at https://explore.plos.org/phishing.

If we can help with anything else, please email us at customercare@plos.org.

Thank you for submitting your work to PLOS ONE and supporting open access.

Kind regards,

PLOS ONE Editorial Office Staff

on behalf of

Professor Jie Zeng

Academic Editor

PLOS One
